# Hypervalent iodine-mediated intramolecular alkene halocyclisation

**DOI:** 10.3762/bjoc.20.258

**Published:** 2024-11-28

**Authors:** Charu Bansal, Oliver Ruggles, Albert C Rowett, Alastair J J Lennox

**Affiliations:** 1 University of Bristol, School of Chemistry, Bristol, BS8 1TS, UK

**Keywords:** cyclisation, halogenation, heterocycles, hypervalent iodine, oxidation

## Abstract

The chemistry of hypervalent iodine (HVI) reagents has gathered increased attention towards the synthesis of a wide range of chemical structures. HVI reagents are characterized by their diverse reactivity as oxidants and electrophilic reagents. In addition, they are inexpensive, non-toxic and considered to be environmentally friendly. An important application of HVI reagents is the synthesis of halogenated cyclic compounds, in particular, the intramolecular HVI-mediated halocyclisation of alkenes using carbon, oxygen, nitrogen or sulfur nucleophiles. Herein, we describe the examples reported in the literature, which are organised by the different halogens involved and the internal nucleophiles.

## Introduction

**Figure 1:**
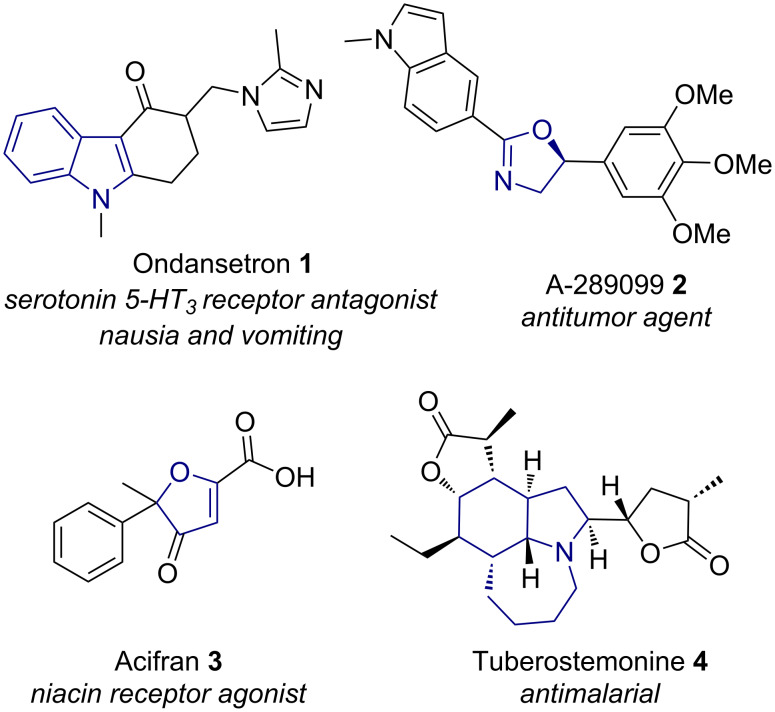
Example bioactive compounds containing cyclic scaffolds potentially accessible by HVI chemistry.

Halogenated carbocyclic and heterocyclic compounds are present in many active pharmaceutical ingredients [1–2]. The intramolecular halocyclisation of alkenes mediated by HVI(III) reagents allow access to a range of halogenated cyclic scaffolds in a cost effective and selective, one-pot transformation. Pharmaceutical uses for bio-active cyclic molecules accessible by I(III) reagents are plentiful; anticancer drugs can be formed from the basis of pyrrolo[2,3-*b*]indoles **1** [3–4], 2-oxazolines **2** [5–6], dihydrofuran **3** [7–8], and spirocyclic scaffolds **4** [9–10] (Figure 1). Halogenated cyclised structures have also been found to exhibit medicinal and pharmaceutical properties, including antibacterial [11], antibiotic [12], and enzyme inhibition [13] among others.

**Figure 2:**
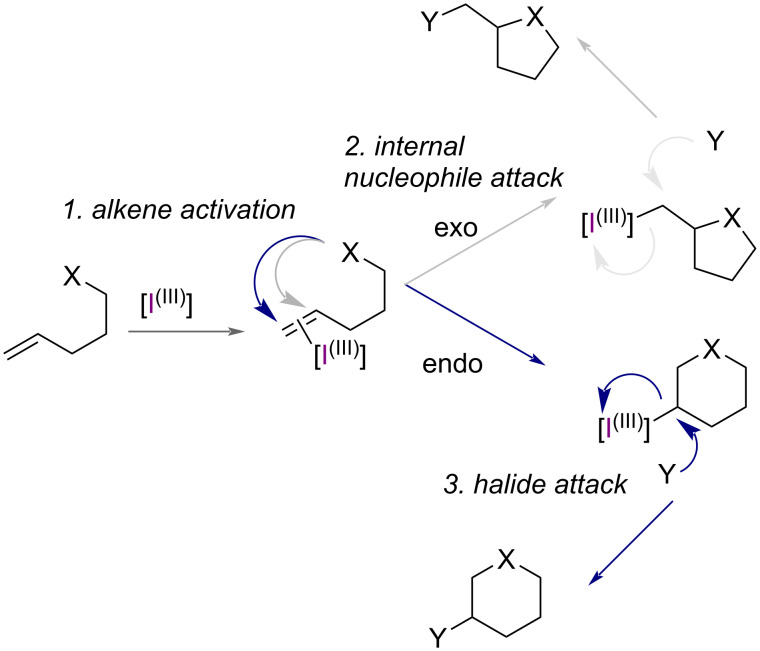
A general mechanism for HVI-mediated e*ndo*- or *exo*-halocyclisation.

The general mechanism for the HVI-mediated halocyclisation of alkenes proceeds firstly through the coordination of an alkene by the HVI reagent, which activates it toward intramolecular attack by an internal nucleophile. Following this, substitution of the iodane(III) can occur from the nucleophilic halide in solution to reveal the halo-cyclised product (Figure 2).

In this review, we have collected and described the HVI-mediated halocyclisation reactions reported in the literature for each halide. We have organised the examples firstly by the halide nucleophile, and then secondly by the internal heteroatom nucleophile involved in the cyclisation. The selectivity (chemo-, regio-, stereo-) of the reaction, which is influenced by the type of HVI reagent, the nature of the substrates employed and the proposed mechanism from the authors are all described. The halocyclisation of alkenes to make halogenated carbo or heterocycles is yet to be covered by a review, which is the vacancy this review aims to fill. The synthetic uses of HVI reagents [14–16], their involvement in heterocycle synthesis [17–19], and alkene functionalisation [20–21], have each been well-reviewed elsewhere.

## Review

### Hypervalent iodine-mediated fluorocyclisation

Fluorine can substantially improve the activity of biologically relevant molecules [22], and compounds containing fluorine have seen huge success in medicine and agrochemicals, with over 30% of small molecule drugs [23–24] and 16% of pesticides [25] now containing fluorine atoms. A range of synthetically important fluorinated hetero- and carbocycles can be synthesized mildly and effectively using HVI reagents.

#### Nitrogen nucleophiles

**Scheme 1:**
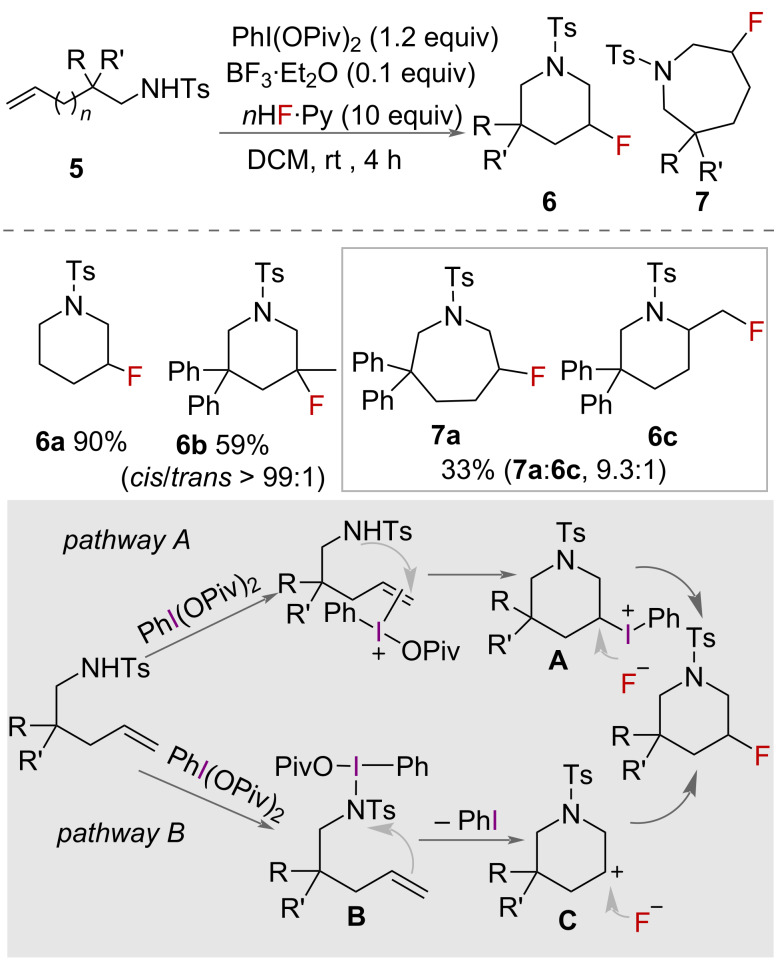
Metal-free synthesis of β-fluorinated piperidines **6**. Ts = tosyl.

A metal-free synthesis of β-fluorinated piperidines was reported in 2012 by Meng, Li and co-workers (Scheme 1) [26]. The authors describe a reaction using PhI(OPiv)_2_ as oxidant with HF·pyridine as the source of fluoride and BF_3_·OEt_2_ as activator. A range of unsaturated amines **5** were cyclised to racemic β-fluorinated piperidines **6**. Good yields were reported for all compounds except those with substituents present on the alkene. Homologation of the carbon chain from 5 to 6 carbons gave both 6- and 7-membered rings in poor yield with α preference for 7-membered rings **7** in a ratio of 1:9.3.

The authors proposed two mechanisms for the reaction (Scheme 1). In pathway A (top), the alkene-activated iodonium is formed, intramolecular attack of nitrogen forms the 6-membered ring **A** before an S_N_2 reaction with the fluoride ion to displace PhI. In pathway B (bottom), the nitrogen is oxidised by the iodane, generating an electrophilic intermediate **B**. Nucleophilic attack by the double bond subsequently forms the 6-membered ring intermediate **C**, which is either immediately attacked by fluoride to form both *cis* and *trans* products or stabilised by the tosyl group and subsequently attacked to form only the *cis* product in an S_N_2 reaction.

**Scheme 2:**
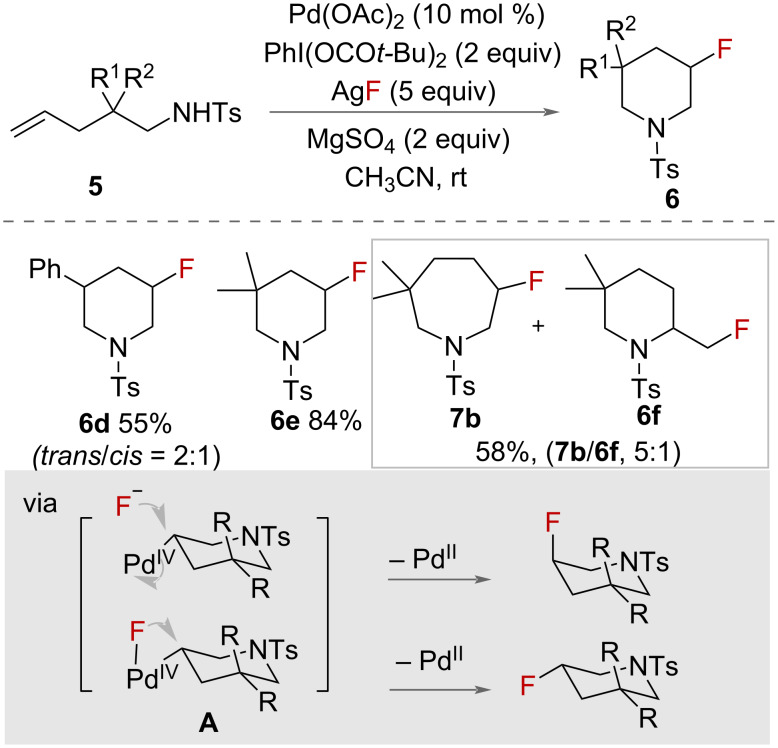
Intramolecular aminofluorination of unactivated alkenes with a palladium catalyst.

Liu and co-workers reported a palladium-catalysed intramolecular aminofluorination of unactivated alkenes [27] (Scheme 2) in the presence of PhI(OPiv)_2_, AgF and MgSO_4_ as an oxidant, source of fluorine and additive, respectively. Racemic β-fluorinated piperidines **6** were synthesised in excellent yields, under mild conditions. A small amount of amino carboxylation side-product was determined to have been additionally produced from the reaction. A range of other alkenes were cyclised in good yields, demonstrating the scope of the reaction. The authors proposed an alternative reaction mechanism to those already described, in which *trans*-aminopalladation of the alkene, mediated by Pd(II), occurs with intramolecular attack of the nitrogen on the terminal carbon, generating a 6-membered ring **A** (Scheme 2). The Pd(II) intermediate is oxidised by PhI(OPiv)_2_/AgF, forming Pd(IV). Formation of the product can occur either by reductive elimination by Pd(IV) or S_N_2 nucleophilic attack by fluorine with concomitant palladium reduction. Reductive elimination of the Pd(II) intermediate forms the C‒F bond to give predominantly the *trans* product, but this pathway competes with a less favourable S_N_2 nucleophilic attack by fluorine to form the *cis* product. However, a mechanism entirely mediated by the I(III) HVI reagent, with the Pd(OAc)_2_ only acting as a Lewis acid to activate the HVI reagent, was not ruled out.

**Scheme 3:**
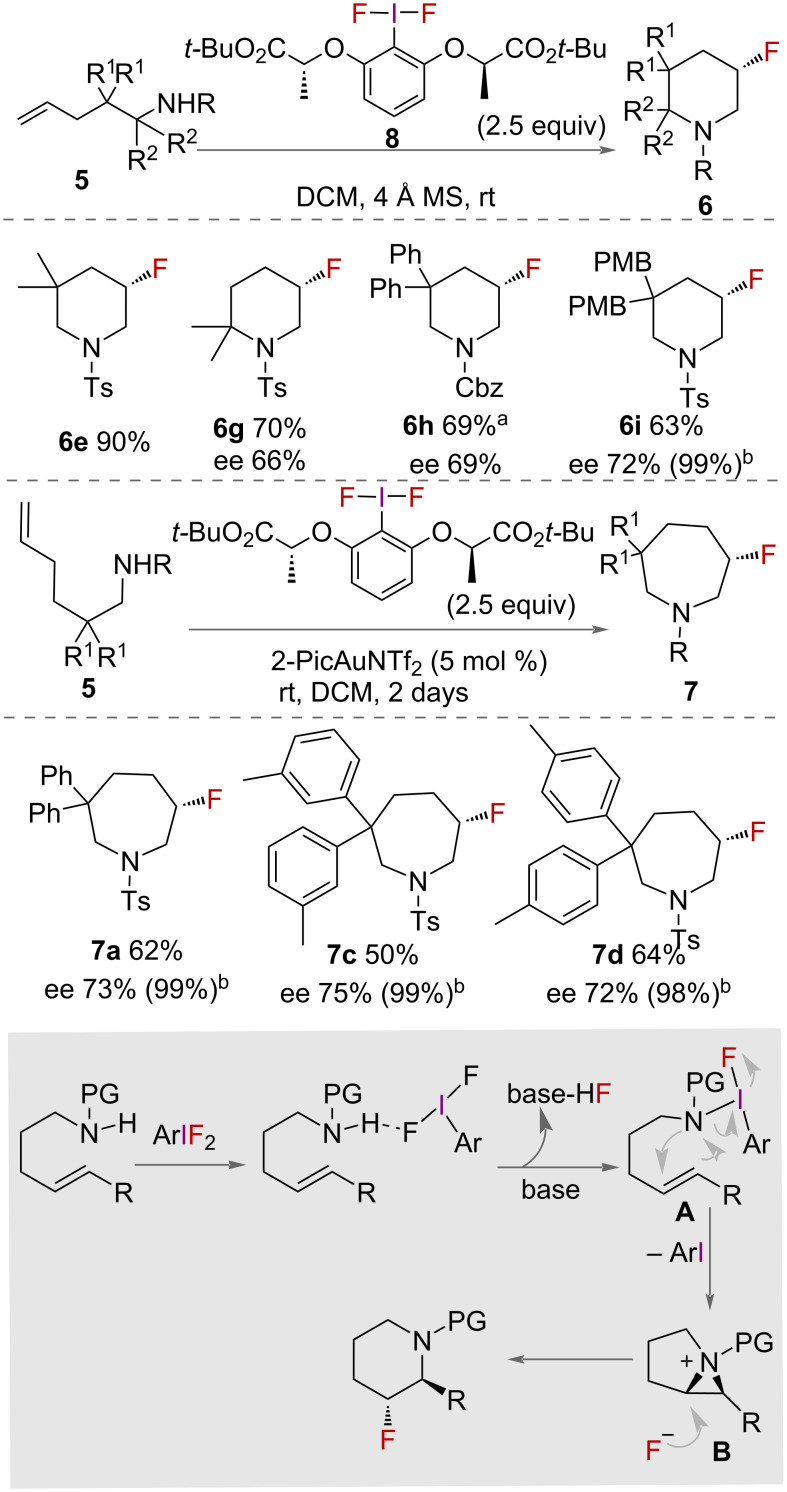
Aminofluorination of alkenes in the synthesis of enantiomerically pure β-fluorinated piperidines. PMB = *para*-methoxybenzene, ^a^the ee value was determined after Cbz group was replaced with tosyl group, ^b^the value in brackets corresponds to the ee value after crystallization. PG = protecting group.

In 2013, Nevado and co-workers used (*R*,*R*)- and (*S*,*S*)-*tert*-butyl lactate iodotoluene difluoride (**8**) for the aminofluorination of alkenes toward the synthesis of enantiomerically pure β-fluorinated piperidines **6** (Scheme 3) [28]. A range of enantiomerically pure fluorinated piperidines were synthesised in moderate to excellent yields and enantiomeric excesses. In addition to the synthesis of 6-membered rings, 7-membered hexenamines **7** were synthesised. To the authors’ surprise, reaction conditions for the synthesis of β-fluorinated piperidines **6** afforded no product. The reaction instead proceeded with addition of dichloro(pyridine-2-carboxylato)gold(III) complex in combination with silver triflimide, AgNTf_2_. A range of β-fluoroazepanes **7** were successfully synthesised with high enantiomeric purity in good yields. A mechanism for the synthesis of β-fluorinated piperidines was proposed by the authors (Scheme 3). Activation of the HVI reagent by H-bonding leads to ligand exchange to give an aminofluoro iodonium intermediate **A**. Cyclisation occurs via nitrogen attack on the alkene to then give aziridinium intermediate **B**. Subsequent nucleophilic attack by fluoride on the more substituted carbon that is more cationic leads to the product.

**Scheme 4:**
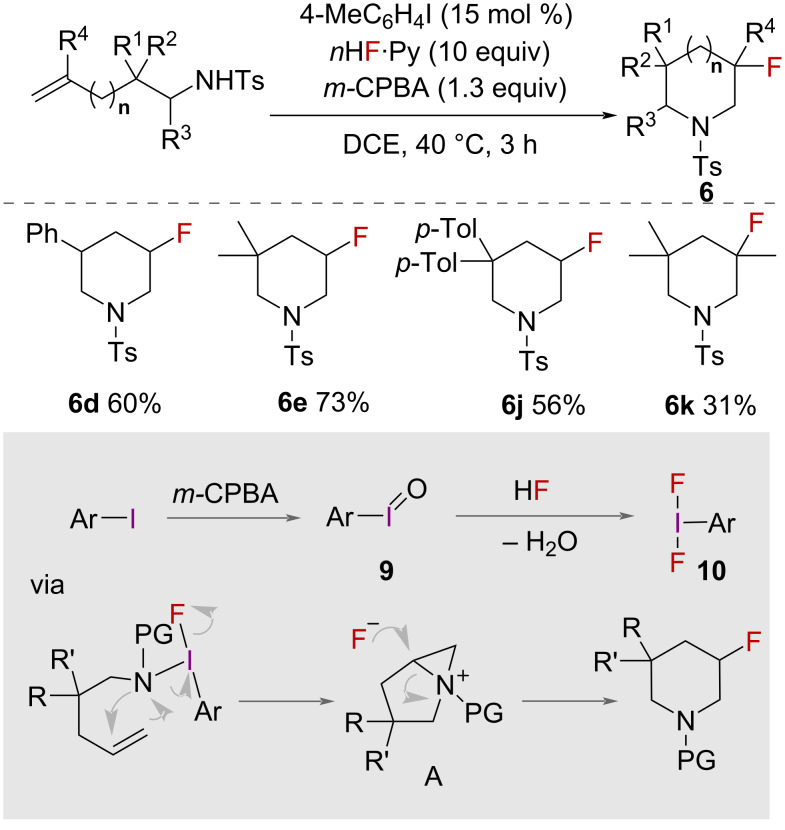
Synthesis of β-fluorinated piperidines.

Synthesis of β-fluorinated piperidines **6** with in situ-generated HVI reagent was reported in 2014 by Kita, Shibata and co-workers (Scheme 4) [29]. Using difluoroiodotoluene **10**, formed in situ from 4-iodotoluene, pyridine·HF and *m*-CPBA, intramolecular aminofluorination of a range of unsaturated amines formed β*-*fluorinated piperidines **6** and 3-fluoroazepanes **7** in good yields. Again, yields only significantly fell with substrates containing substituents on the alkene. Depending on the length of the alkyl chain, both 6- and 7-membered rings were formed. The use of a chiral aryl iodide was tested, which gave products with low enantiomeric excess. However, these preliminary trials represent the first example of a catalytic, enantioselective HVI-mediated fluorocyclisation.

The authors proposed a mechanism (Scheme 4) for this reaction that involved iodoarene difluoride **10** being generated from iodosylarene **9** (ArI=O) and HF, with iodosylarene itself generated by aryl iodide and *m-*CPBA. Ligand exchange of iodoarene difluoride with nitrogen and reaction with the alkene forms aziridinium intermediate **A** which, after nucleophilic attack by fluoride, forms the product.

**Scheme 5:**
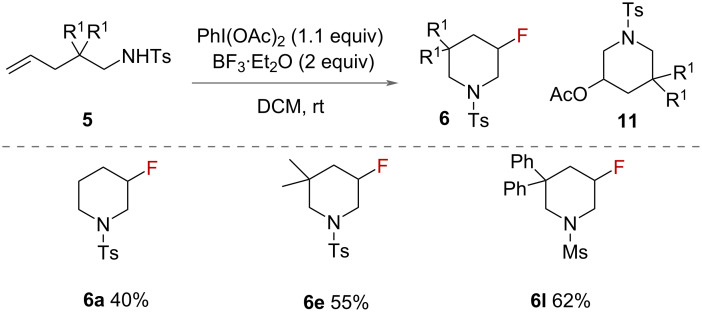
Intramolecular fluoroaminations of unsaturated amines published by Li.

Li reported a haloamination of unsaturated amines in 2014 (Scheme 5) [30], to form fluorinated piperidines **6** using PhI(OAc)_2_ as an oxidant and BF_3_·OEt_2_ as the source of fluoride. Fluorocyclisations gave lower yields compared to other halocyclisations reported by the authors. Aminoacetylation of the alkene competed with the aminofluorination to form 3-acetoxypiperidines **11**. Other sources of fluoride were tested, with metal fluoride salts giving no or trace products. The authors reported that only 6-membered rings were formed, with a range of substituents on the β-carbon of the alkene. The authors proposed a mechanism for the reaction, in which the alkene is activated by PhI(OAc)_2_, followed by intramolecular nucleophilic attack of nitrogen and displacement of iodobenzene by fluoride.

**Scheme 6:**
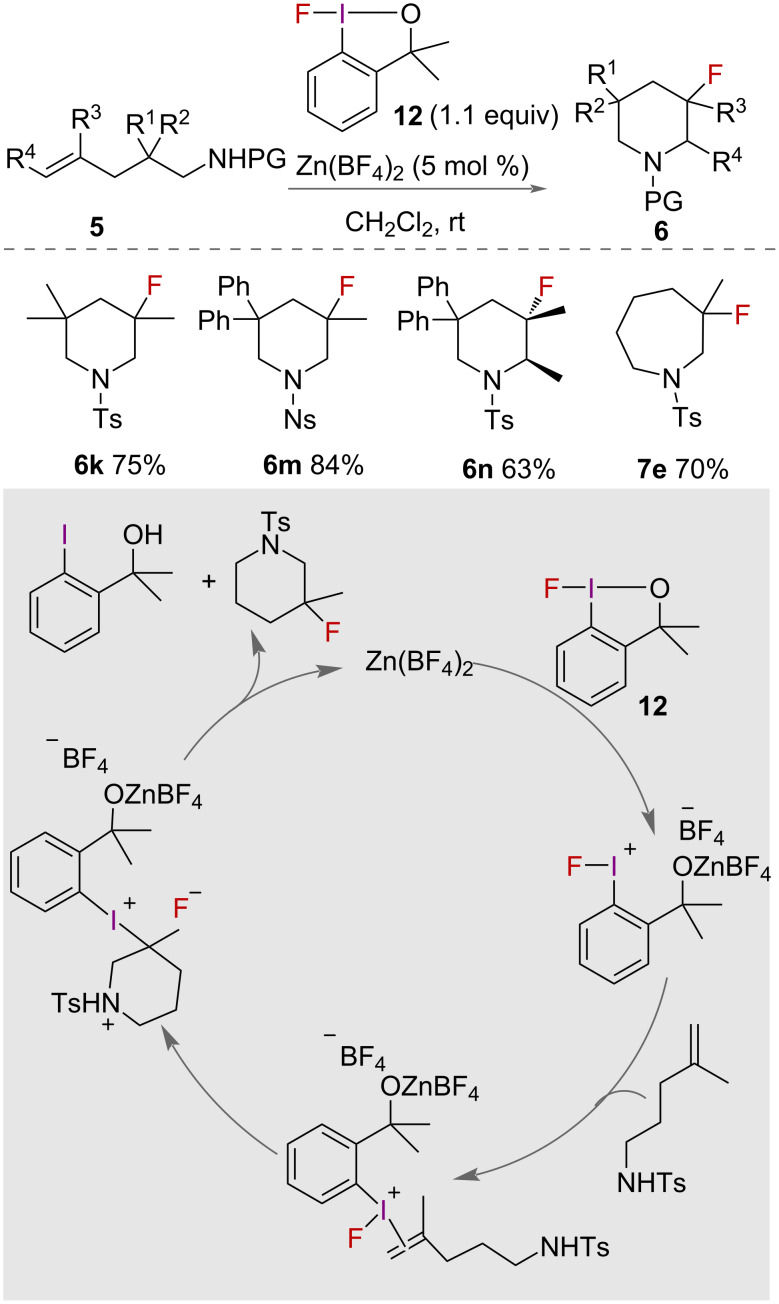
Intramolecular aminofluorination of unsaturated amines using 1-fluoro-3,3-dimethylbenziodoxole (12). PG = protecting group.

The intramolecular aminofluorination of unsaturated amines using a structural analogue of Togni’s reagent, 1-fluoro-3,3-dimethylbenziodoxole (**12**), was reported in 2015 by Szabó (Scheme 6) [31]. With catalytic Zn(BF_4_)_2_, 1-fluoro-3,3- dimethylbenziodoxole mediated the formation of fluorinated piperidines **6** and hexanamines **7** in high yields. A range of unsaturated amines with substituents on both carbons of the alkene were successfully cyclised in similarly good yields. However, increased substitution on the alkene resulted in an increased reaction time, with 9 hours required for disubstituted alkenes compared to 3 hours for non-substituted alkenes. With extended chain lengths, 5-, 6- and 7-membered aza-heterocycles were synthesised in good yields.

The authors proposed a mechanism for the fluorocyclisation reactions (Scheme 6), which relies on the activation of the fluoro-iodane reagent **12** with the zinc catalyst. The activation enables better orbital overlap to occur with the π bond of the alkene to form an iodonium species. Nucleophilic attack occurs on the least hindered carbon of the iodonium, before displacement of the HVI by fluoride to give the product.

**Scheme 7:**
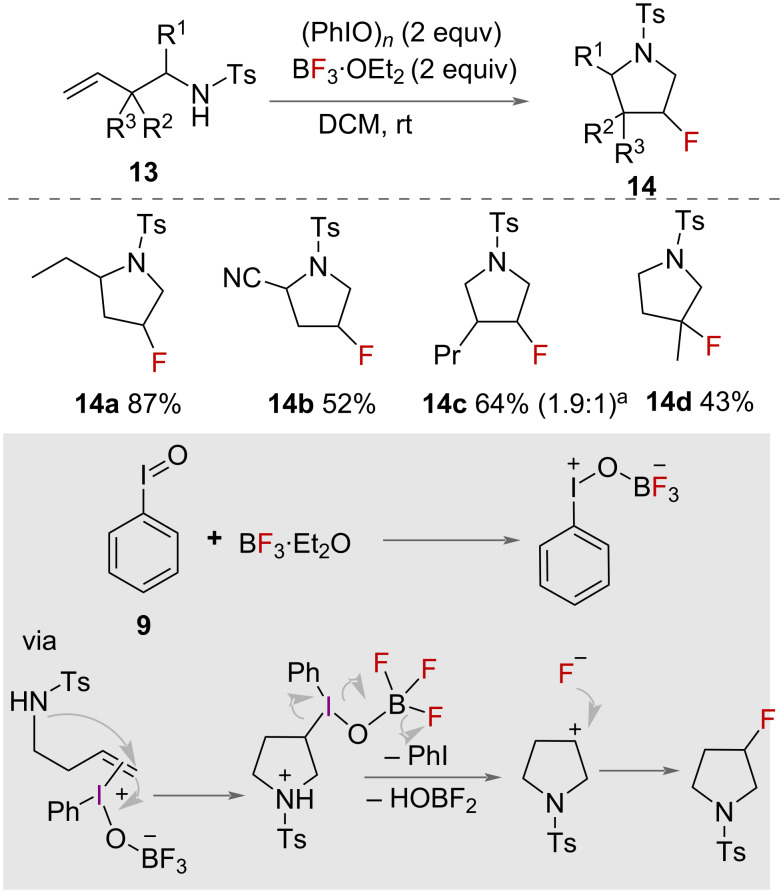
3-fluoropyrrolidine synthesis. ^a^Diastereomeric ratio (*cis*/*trans*) determined by ^19^F NMR analysis.

Zhang and co-workers reported the intramolecular aminofluorination of unsaturated amines using an HVI reagent generated in situ from iodosylbenzene **9** (Scheme 7) [32]. The authors reported the synthesis of 3-fluoropyrrolidines **14** with BF_3_·OEt_2_ as a source of fluoride for the intramolecular aminofluorination of homoallylic amines **13**. The authors explored the reaction with various protecting groups on nitrogen and substituents on the alkene and alkyl chain. Substrates with *p*-tolylsulfonyl (Ts), *p*-nitrobenzenesulfonyl (Ns) and benzenesulfonyl (Bs) protecting groups were cyclised in high yields to the corresponding 3-fluoropyrrolidine derivatives **14**. A range of unsaturated amines were successfully cyclised. However, substrates with substituents on the alkene gave low yields of product. A mechanism was proposed involving the activation of iodosylbenzene **9** with BF_3_·Et_2_O to form an HVI intermediate that activates the alkene to form an iodonium species. Intramolecular nucleophilic attack of nitrogen, elimination of PhI and attack by fluoride then forms the product.

**Scheme 8:**
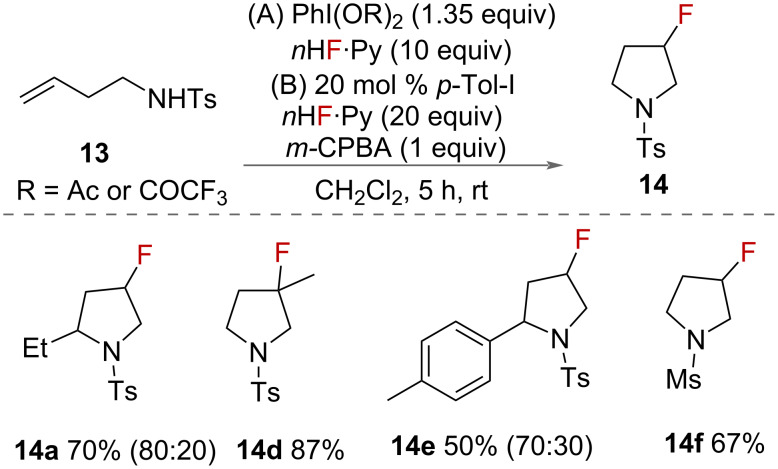
Kitamura’s synthesis of 3-fluoropyrrolidines. Values in parentheses represent the *cis*:*trans* ratio.

Kitamura and co-workers additionally reported a synthesis of 3-fluoropyrrolidines **14** in 2017 using an alternative source of fluoride (Scheme 8) [33]. The authors used either PhI(OAc)_2_ or PhI(OCOCF_3_)_2_ as oxidants and pyridine·HF as a source of fluoride. This alternative reagent system was used due to concerns with the long-term stability of iodosylbenzene and unwanted reactions of BF_3_·Et_2_O with other reagents. In addition, a catalytic system was reported that employed 20 mol % iodotoluene with 1 equivalent of *m*-CPBA as terminal oxidant. The authors proposed that difluoroiodobenzene **10** is formed in situ, which is activated by HF. Two possible mechanisms were given for the synthesis of pyrrolidines **14**, which are the same two proposed for the synthesis of piperidines **6** (Scheme 1). Either an alkene-activated iodonium is formed or an activated electrophilic nitrogen is generated from interaction with the HVI, that is then attacked by the alkene.

**Scheme 9:**
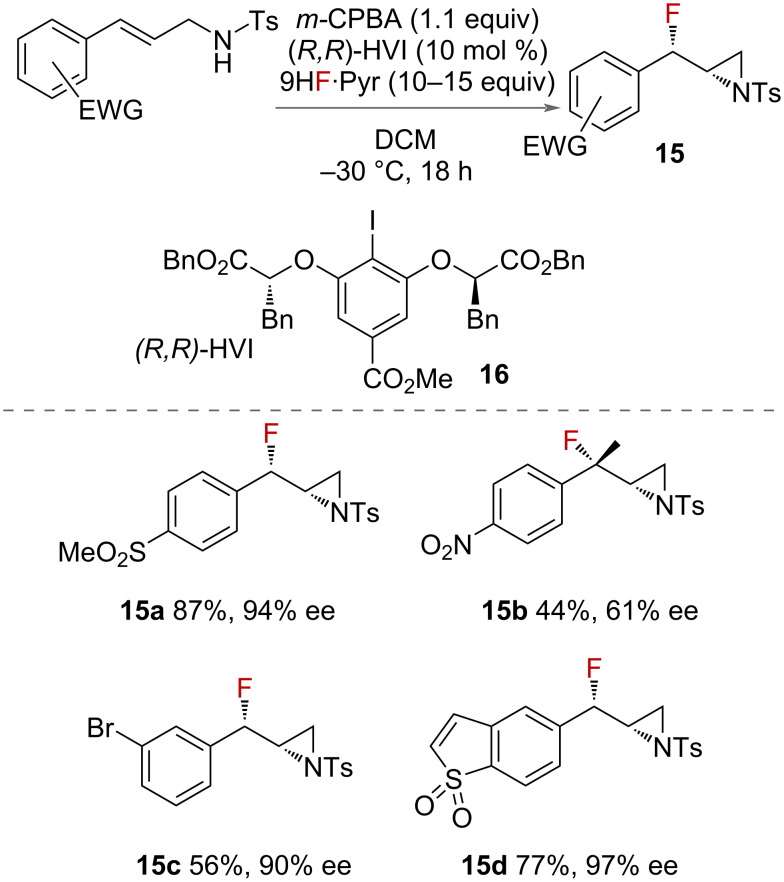
Jacobsen’s enantio- and diastereoselective protocol for the synthesis of *syn*-β-fluoroaziridines **15**.

While the fluoroamination of alkenes to form 4-membered azetidines has not been reported, an *exo* fluorocyclisation to form 3-membered aziridines with an adjacent fluorine was reported by Jacobsen in 2018 (Scheme 9) [34]. The synthesis from the styrenyl starting materials is stereoselective, giving the *syn*-diasteroisomer in high yields. A chiral iodoarene catalyst **16** was employed, along with a stoichiometric sacrificial oxidant, to give good to excellent levels of enantioselectivity. This elegant strategy led to a variety of β-fluorinated tosylated aziridines **15** with good tolerance to styrenyl arenes with electron-withdrawing groups on them. The yields of product dropped off significantly when the ring did not contain an electron-withdrawing group on it.

**Scheme 10:**
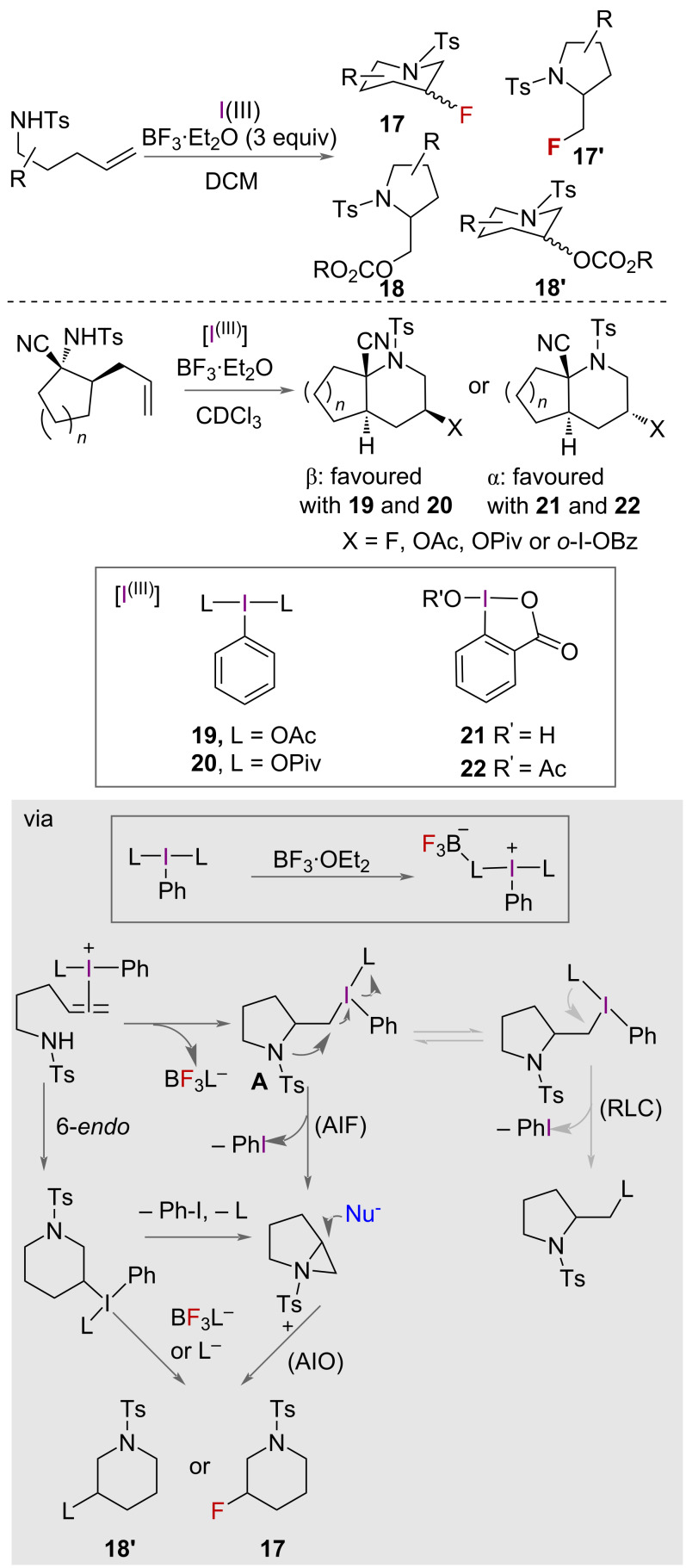
Different HVI reagents lead to different diastereoselectivity in aminofluorination competing with carboxyfluorination products. Mechanistic studies of the fluoroamination of acyclic substrates showing the competing carboxyfluorination products. AIF = aziridinium ion formation, AIO = aziridinium ion opening, RLC = reductive ligand coupling.

In 2023, Šmit and co-workers conducted an in-depth mechanistic study on the cyclisation of alkenyl *N*-tosylamides using BF_3_-activated aryl iodane(III) carboxylates to create 3-fluoropiperidines [35]. The challenges faced relate to selectivity due to competing carboxyaminations (**18**, **18’**), rather than fluoroamination (**17**, **17’**), and difficulties in controlling the diastereoselectivity. The authors studied the stereo-, regio, and chemoselectivity in both cyclic and acyclic substrates. It was observed that the use of acyclic iodane reagents **19** and **20** predominantly led to products with β-stereochemistry, whereas the cyclic iodanes **21** and **22** favour pathways leading to *α*-stereochemistry (Scheme 10). The selectivity of the reaction was also found to be influenced by the presence of electrolytes like TBABF_4_, and the ligand attached to I(III). Carboxyfluorination was observed, in which a ligand from the iodane, e.g., OAc, OPiv or *o*-I-OBz, adds and was found to compete with fluoroamination. The level of this chemoselectivity was dependent on the iodane ligand: OPiv was more selective for aminofluorination than OAc, which was proposed to be due to differences in basicity and nucleophilicity (Scheme 10).

Detailed mechanistic studies were carried out using multinuclear NMR spectroscopy, deuterium labelling, rearrangements on stereodefined substrates, and structural analyses (NMR and X-ray) of the reaction products. RT-NMR-derived data strongly supported a pathway of alkene activation by the iodane, as opposed to the formation of an N-I(III) adduct. The presence of 5-*exo*-products, with support of a deuterium labelling experiment, also ruled out the possibility of an *N*-activation pathway. Therefore, the proposed mechanism involves BF_3_-coordinated I(III) iodane forming iodiranium(III) ions with the alkene, followed by diastereo-determining 5-*exo*-cyclisation. These transiently formed pyrrolidine intermediates **A** can undergo further transformations (Scheme 10), depending on their structure and the iodane reagent being used. For example, higher yields of 3-fluoropiperidine products **6** were observed when using cyclic iodane reagents **21** and **22** (Scheme 10), which was suggested to be because a reductive ligand coupling (RLC) pathway would be suppressed due to reduced fluxionality of the carboxylate ligand on I(III). These important findings are expected to enhance the use of aryl iodane(III)-dicarboxylates for constructing fluorinated azaheterocycles with improved selectivity and control.

#### Oxygen nucleophiles

**Scheme 11:**
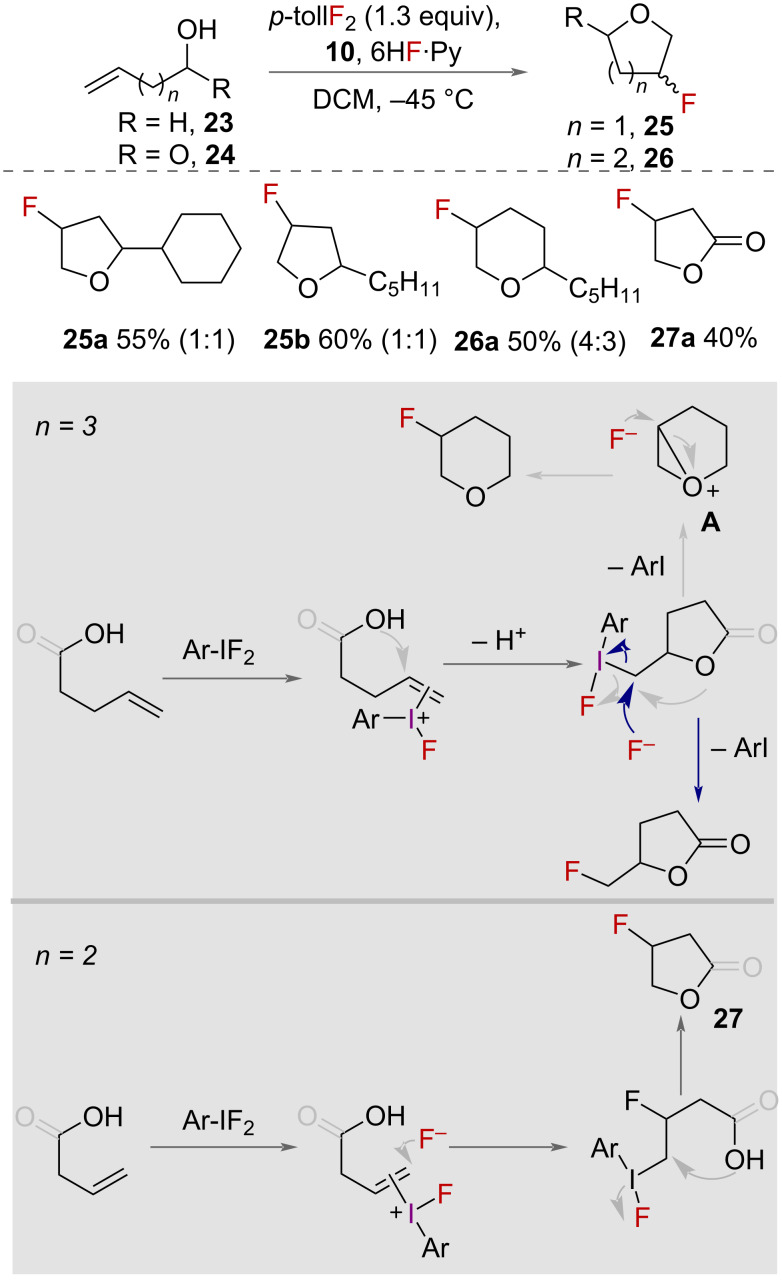
Fluorocyclisation of unsaturated alcohols and carboxylic acids to make tetrahydrofurans, fluoromethyl-γ-lactones and tetrahydropyrans. The ratio of stereoisomers is shown in parentheses.

In 2000, Hara and co-workers reported the fluorocyclisation of unsaturated alcohols and carboxylic acids promoted by HVI reagents (Scheme 11) [36]. Using 4-tolyl difluoroiodane **10** as the reaction promoter, and pyridine·6HF as a source of fluoride, a range of unsaturated alcohols **23** to fluorinated tetrahydrofurans **25** and tetrahydropyrans **26**. Unsaturated carboxylic acids **24** was also cyclised to form 5-membered fluorinated lactones **27**.

The mechanisms proposed for the cyclisation of unsaturated alcohols and carboxylic acids both proceed first through the activation of the alkene by the iodane (Scheme 11). The internal oxygen nucleophile and fluoride then sequentially attack the activated alkene, to either form 5- or 6-membered furan or pyran heterocycles depending on the chain length in the substrate. The pyran ring is only formed from the alcohol starting material, presumably because the oxygen is reactive enough to displace the iodane to form the oxonium species **A**. When the carboxylic acid is used, the oxygen in the lactone intermediate is less reactive and so substitution of the iodane by fluoride is more favourable and the branched product is formed.

**Scheme 12:**
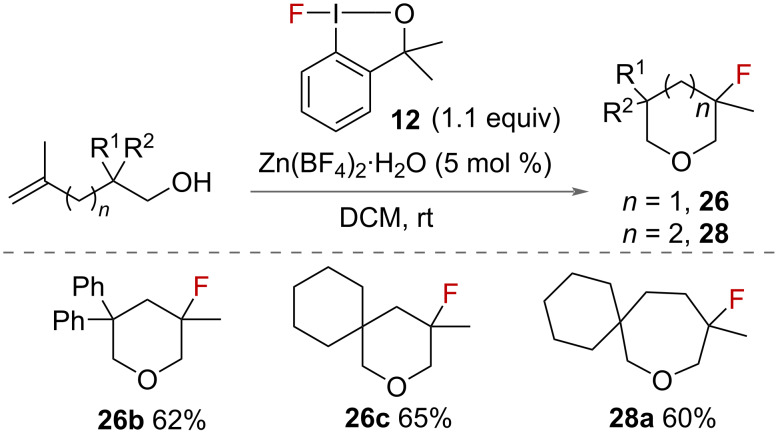
Oxyfluorination of unsaturated alcohols.

In addition to aminofluorination, Szabó also reported the oxyfluorination of alkenes in 2015 [31]. Under identical conditions to the aminofluorination using 1-fluoro-3,3-dimethylbenziodoxole (**12**) with Zn(BF_4_)_2_ catalyst, unsaturated alcohols were cyclised to fluorinated tetrahydropyrans **26** and oxepanes **28** (Scheme 12) in 1–2 hours in good yields.

**Scheme 13:**
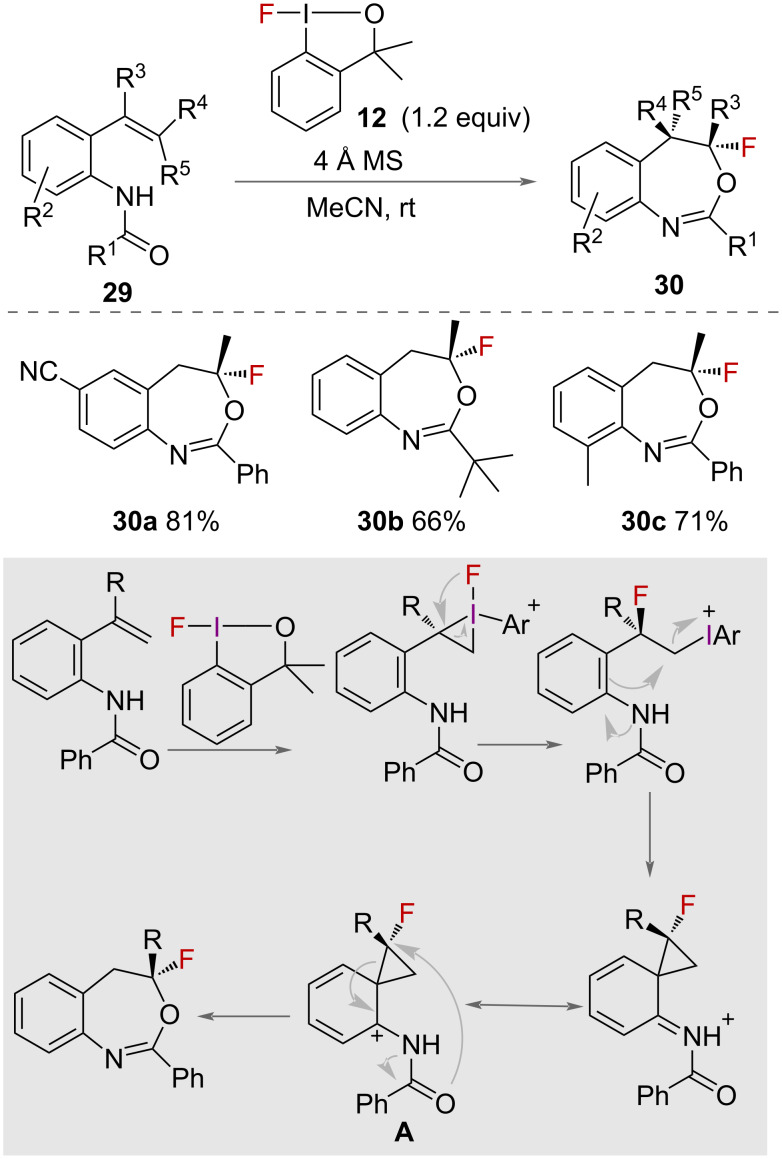
Synthesis and mechanism of fluoro-benzoxazepines.

Gulder and co-workers reported a mild, metal free-synthesis of fluorobenzoxazepines **30** in 2016 (Scheme 13) [37]. Using 1-fluoro-3,3-dimethylbenziodoxole (**12**) and 4 Å molecular sieves, a range of benzamides **29** were successfully cyclised in good yields. The sustainability of the reaction was improved by regeneration of 1-fluoro-3,3- dimethylbenziodoxole (**12**) in 91% yield from isolated benzyl alcohol after the fluorination reaction. A mechanism of the reaction was proposed (Scheme 13) in which the iodane-activated alkene is attacked by fluoride and the aromatic ring, to form a fluorinated phenonium intermediate **A**. The product is formed following a 6-*endo* cyclisation with nucleophilic attack from oxygen to provide the fluoro-benzoxazepines **30**.

**Scheme 14:**
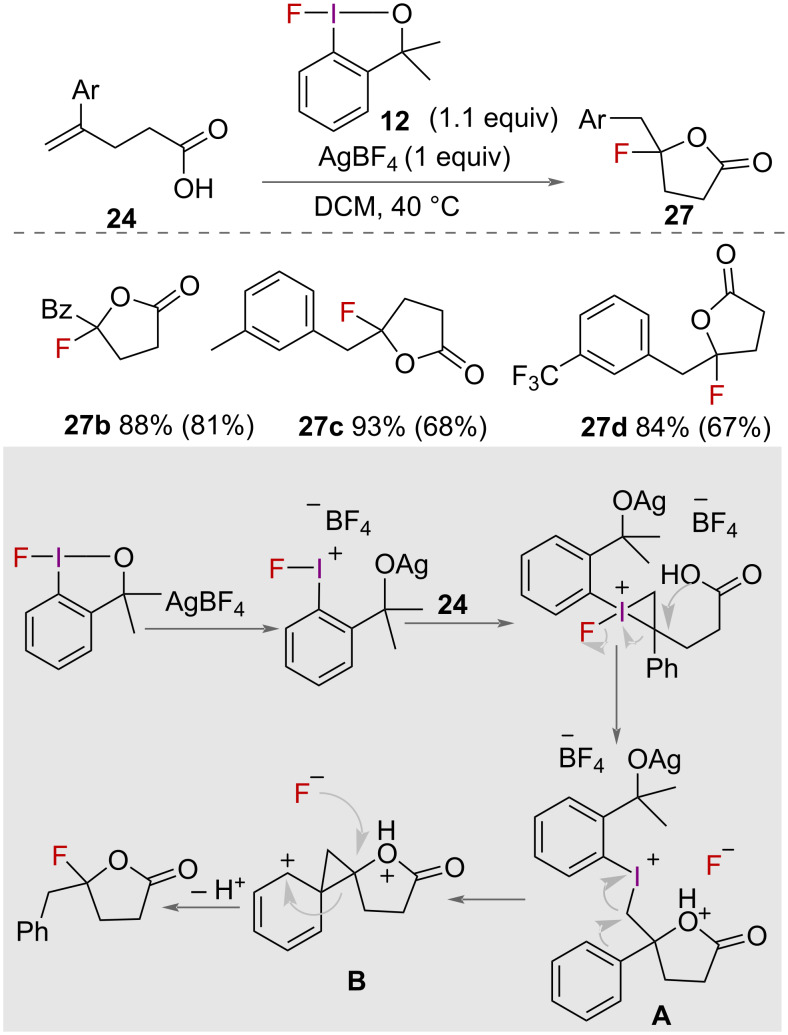
Intramolecular fluorocyclisation of unsaturated carboxylic acids. Yield of isolated product within parentheses.

In 2015, Stuart and co-workers reported an intramolecular fluorocyclisation of unsaturated carboxylic acids **24** (Scheme 14) [38]. Using 1-fluoro-3,3-dimethylbenziodoxole (**12**) as an oxidant with AgBF_4_ and 4 Å molecular sieves to prevent water competing with fluoride as the nucleophile, a range of unsaturated carboxylic acids were successfully cyclised to fluorinated lactones **27** in good yields. The authors proposed a mechanism for fluorolactonization (Scheme 14) whereby AgBF_4_ first activates the fluoroiodane **12** for alkene coordination. Intramolecular nucleophilic attack of oxygen on the more substituted carbon forms the cyclised intermediate **A** and eliminates fluoride. Phenonium intermediate **B** is formed with elimination of the iodoarene and subsequent attack of fluoride forms the product.

**Scheme 15:**
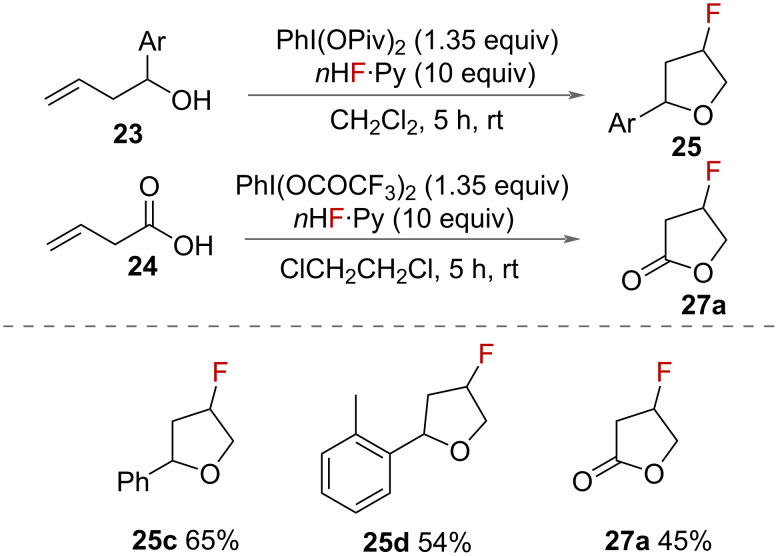
Synthesis of fluorinated tetrahydrofurans and butyrolactone.

In 2017, Kitamura and co-workers reported the synthesis of fluorinated tetrahydrofurans **25** and butyrolactone **27** (Scheme 15) [33]. Unsaturated alcohols **23** and 3-butenoic acid (**24**) were competent starting materials, using PhI(OPiv)_2_ as an oxidant and pyridine·HF as a source of fluoride. Unsaturated alcohols gave moderate yields of racemic fluorinated tetrahydrofurans **26**. Moderate yields were also reported for the fluorocyclisation of 3-butenoic acid (**24**) to form the fluorinated butyrolactone **28**.

**Scheme 16:**
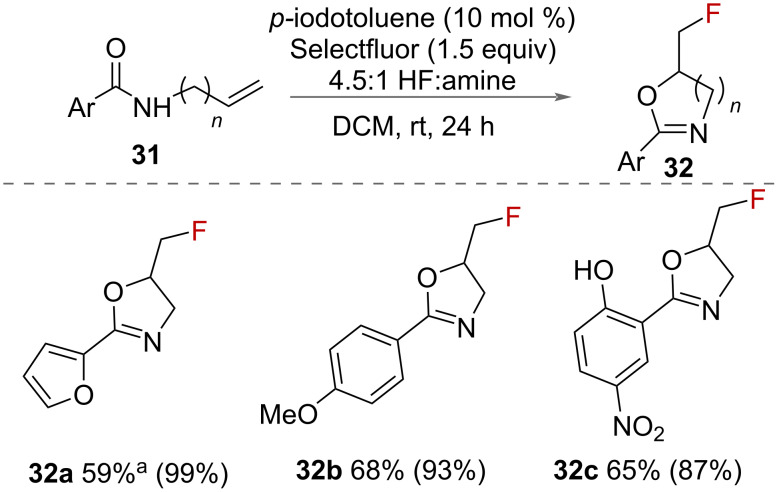
Synthesis of fluorinated oxazolines **32.** ^a^Reaction time increased to 40 hours. Yields refer to isolated values whilst NMR yields are given in parentheses (^19^F NMR using ethyl fluoroacetate as an internal standard).

The preparation of fluorinated oxazolines was reported in 2018 by Gilmour and co-workers (Scheme 16) [39]. *p*-TolIF_2_ is formed in situ from *p*-iodotoluene and Selectfluor in a 4.5:1 HF:amine solution, which is obtained by combining Et_3_N·3HF and pyridine·HF. A range of *N*-allyl carboxamides **31** were successfully cyclised forming fluoromethyl-2-oxazolines **32** in good yields.

**Scheme 17:**
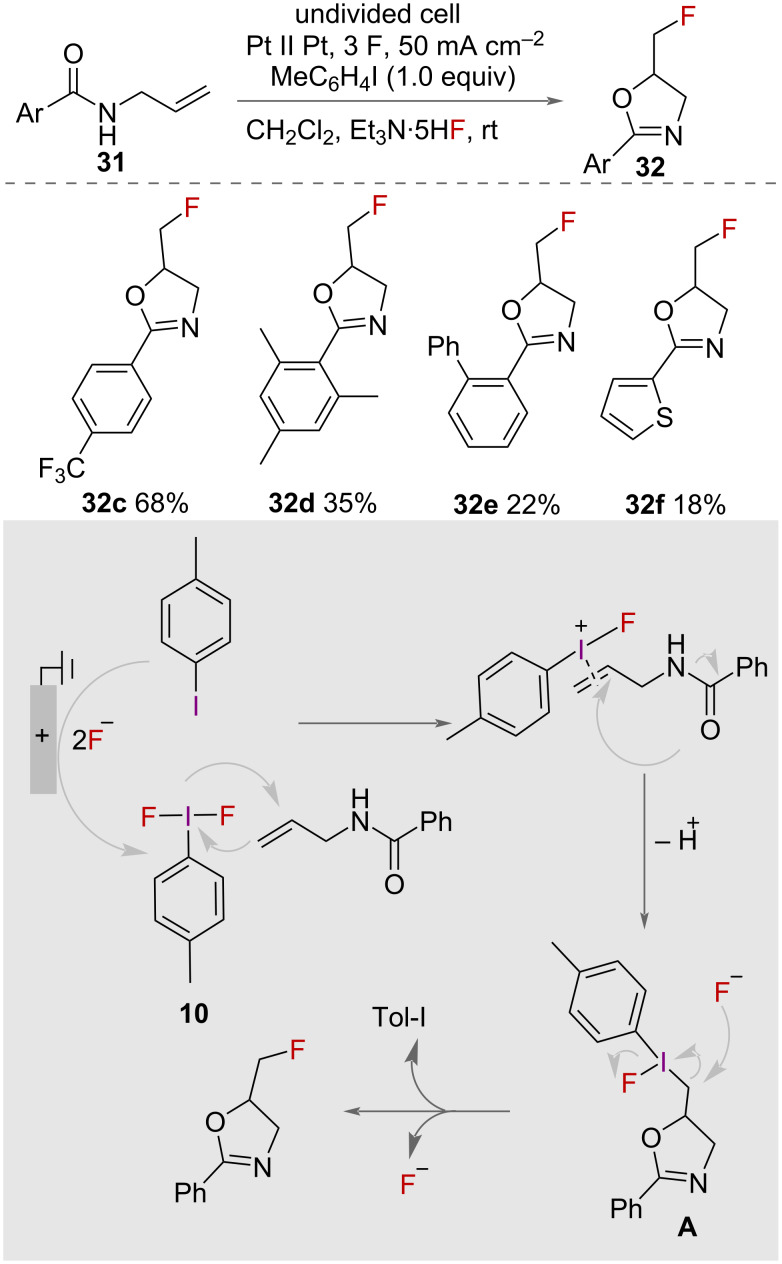
Electrochemical synthesis of fluorinated oxazolines.

The synthesis of fluorinated oxazolines **32** was also reported using an electrochemical approach in 2019 by Waldvogel and co-workers (Scheme 17) [40]. The authors used electrochemical oxidation to form *p*-tolyl-difluoro-λ^3^-iodane **10** on the anode using an undivided cell with platinium electrodes in a 1:1 solution of CH_2_Cl_2_ and Et_3_N·5HF. The in situ formation of this unstable HVI reagent avoided the requirement for it to be isolated. It was used either in a 1-step in-cell procedure with alkene, or in a 2-step, ex-cell approach [41], in which the substrate was added after the electrolysis, thereby avoiding any competing electrochemical oxidation of the substrate. A range of *N*-allylcarboxamides **31** were cyclised to fluorinated oxazolines **32** in moderate to very good yields. Poor yields, however, were reported with electron-withdrawing aryl groups on the substrate. Intramolecular nucleophilic attack from oxygen onto the activated alkene forms the oxazoline **A**, and S_N_2 substitution with the fluoride ion displaces iodotoluene to form the product.

**Scheme 18:**
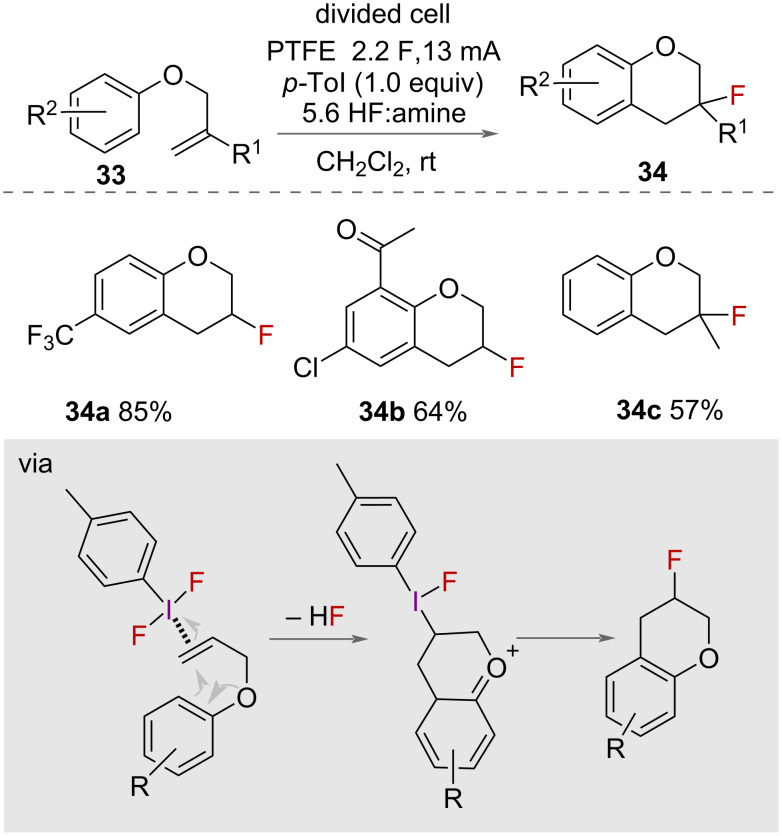
Electrochemical synthesis of chromanes.

An electrochemical approach was also reported by Lennox and co-workers for the synthesis of chromanes **34** (Scheme 18) [42]. The authors reported using *p*-tolyl-difluoro-λ^3^-iodane **10**, formed via electrochemical oxidation of 4-iodotoluene at the anode, in a 5:6 HF:amine mixture to cyclise a range of phenolic ethers **33**. Tolerance for substituents on both the aromatic ring and the alkene were shown, although the electronic requirements were quite narrow for reaction success. As the arene ring attacks the activated alkene, if it is too electron-poor then it is not reactive enough. Moreover, if it is too electron-rich, then it preferentially oxidises via a single electron transfer mechanism which deactivates the ring as a nucleophile.

**Scheme 19:**
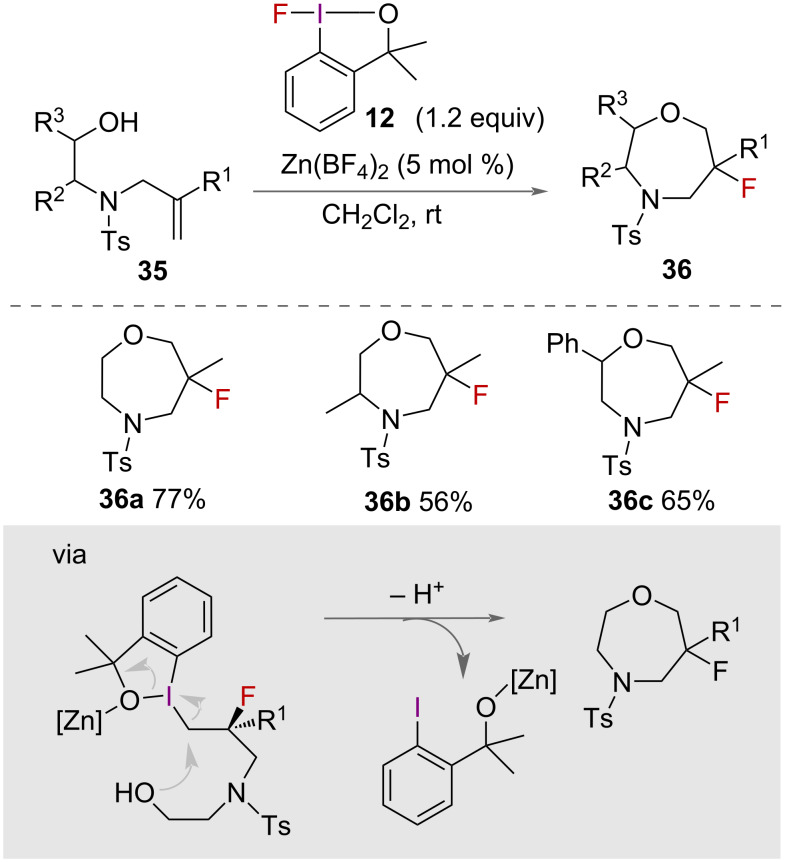
Synthesis of fluorinated oxazepanes.

The synthesis of fluorinated oxazepanes **36** was reported by Ding and co-workers in 2021 (Scheme 19) [43]. Using fluoro benziodoxole **12** as oxidant and Zn(BF_4_)_2_ as a catalytic activator, a range of fluorinated oxazepanes were synthesised from allylamino ethanols **35** in good yields.

**Scheme 20:**
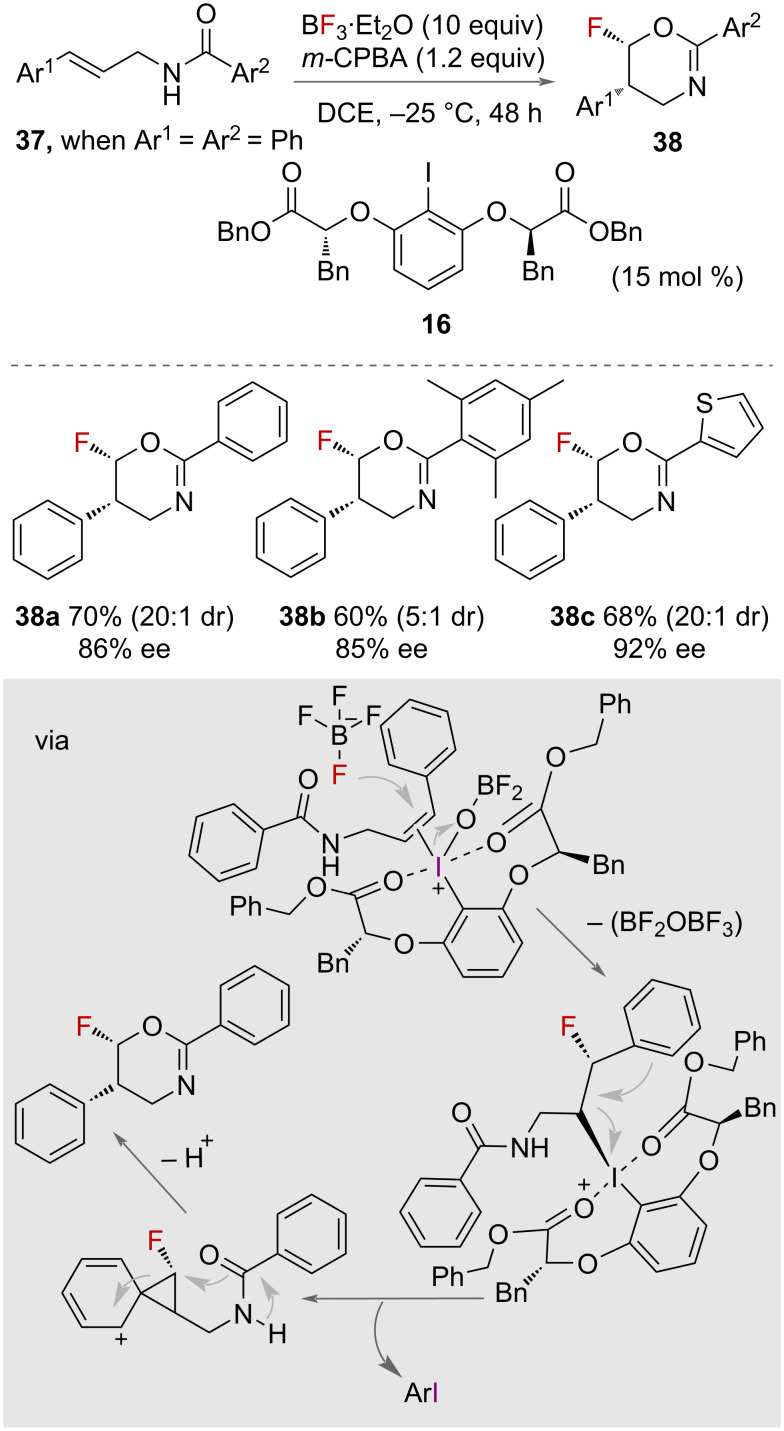
Enantioselective oxy-fluorination with a chiral aryliodide catayst.

In 2021, Jiang and co-workers reported catalytic asymmetric aminofluorination using BF_3_·Et_2_O with a chiral aryliodide **16** catalyst (Scheme 20) [44]. The study successfully obtained various chiral fluorinated oxazine products **38** with high enantioselectivity (up to >99% ee) and diastereoselectivity (up to >20:1 dr). Control experiments showed that using Py·9HF or Et_3_N·3HF as the fluoride source did not yield the desired fluorooxazines, highlighting the role of BF_3_·Et_2_O as both a fluoride reagent and an activating reagent of iodosylbenzene. Different chiral iodide catalysts were studied, revealing that the substituents of the catalysts significantly influenced the stereochemistry of the reaction. Linear chiral catalysts were found to offer higher stereoselectivity compared to spiro-catalysts. Under optimized conditions, the asymmetric aminofluorination of *N*-cinnamylbenzamides **37** using BF_3_·Et_2_O as the fluorine reagent demonstrated good yields and high stereoselectivity (Scheme 20). The scope of the reaction was probed, showcasing the versatility and applicability of the method in synthesizing chiral fluorinated oxazines. The authors proposed the mechanism of the catalytic asymmetric nucleophilic fluorination to involve the activation of iodosylbenzene by BF_3_·Et_2_O which is then attacked by a nucleophile. The use of chiral iodine catalysts is essential for controlling the stereochemistry of the reaction. The specific arrangement of the catalyst influences the orientation of this nucleophilic attack as supported by density functional theory (DFT) calculations.

**Scheme 21:**
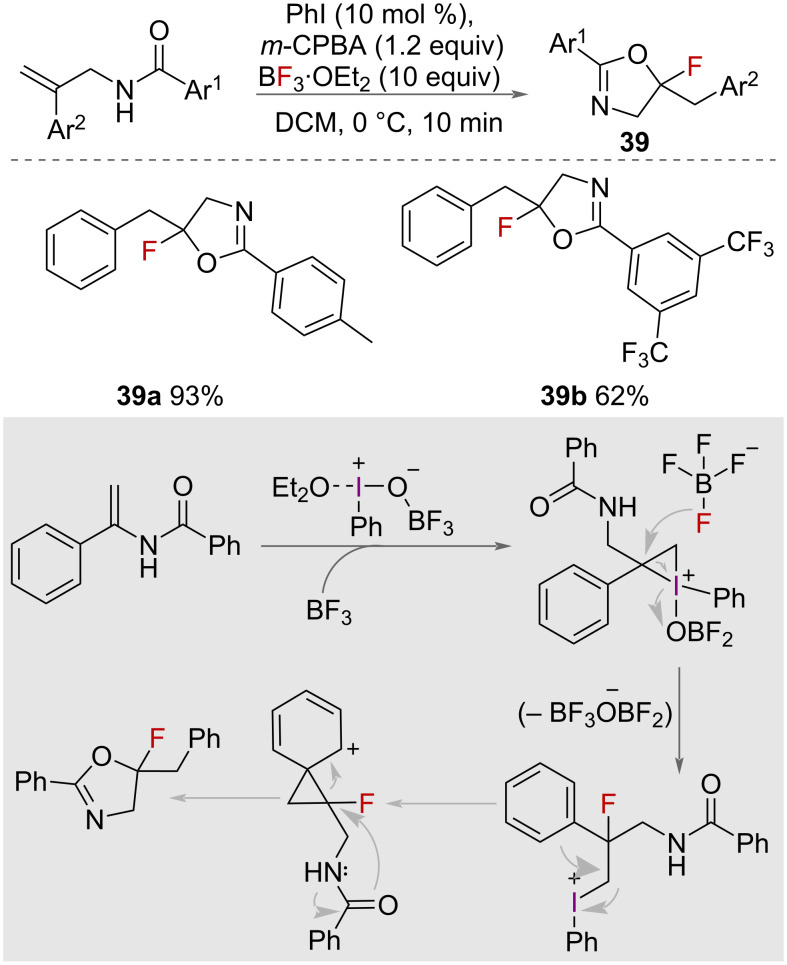
Catalytic synthesis of 5‑fluoro-2-aryloxazolines using BF_3_·Et_2_O as a source of fluoride and an activating reagent.

In 2022, Xu, Zhang, Zhu and co-workers reported a method to catalytically synthesise 5‑fluoro-2-aryloxazolines **39** by utilising BF_3_·Et_2_O as the fluoride source and activating reagent (Scheme 21) [6]. The synthesis of these derivatives was achieved with high efficiency, resulting in good to excellent yields of up to 95% within a short time-frame of 10 minutes. Treatment of *N*-(2-phenylallyl)benzamides with 10 equivalents of BF_3_·Et_2_O, iodobenzene, *m*-CPBA in dichloromethane (DCM) at 0 °C resulted in the formation of the oxazoline product (Scheme 21). DFT calculations indicated several steps in the mechanism, including ligand coupling, oxidative addition, intermolecular nucleophilic attack, 1,2-aryl migration, reductive elimination, and intramolecular nucleophilic attack. This approach offers a rapid and effective way to produce 5-fluoro-2-aryloxazoline compounds, which are valuable building blocks in organic synthesis.

#### Carbon nucleophiles

**Scheme 22:**
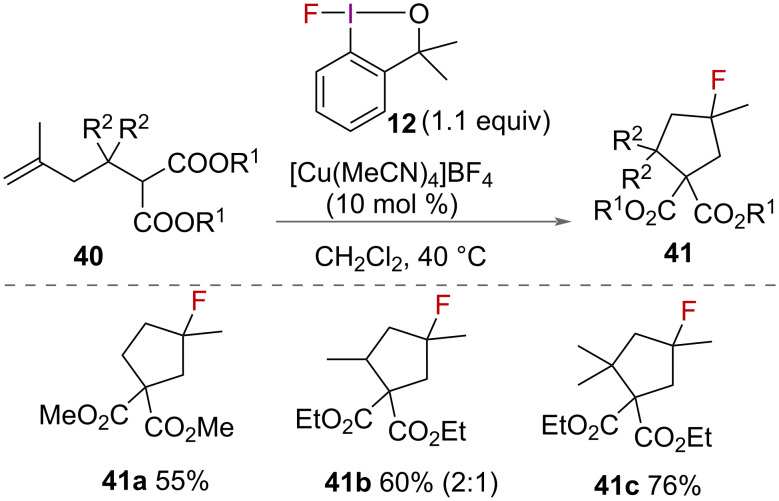
Intramolecular carbofluorination of alkenes.

In addition to intramolecular aminofluorination and oxyfluorination, Szabó and co-workers reported alkene carbofluorination in 2015 (Scheme 22) [31]. Using 1-fluoro-3,3-dimethylbenziodoxole (**12**) and [Cu(MeCN)_4_]BF_4_ as a catalyst to activate it, the authors reported the synthesis of fluorinated cyclopentane products **41** from alkenyl malonate derivatives **40**. The malonate nucleophile required longer reaction times of 8 hours compared to 1–3 hours for aminofluorination and oxyfluorination, however good yields were reported.

### Hypervalent iodine-mediated chlorocyclisation

Although less common than fluorine in biologically active compounds, chlorine-containing molecules have interest in drug discovery, with over 250 chloro-containing drugs presently available [43]. The introduction of a chlorine atom into biologically active compounds for use in pharmaceuticals and agrochemicals has been shown to greatly increase the potency of a compound [45–46] and can be considered a bioisostere for a methyl group. Chlorination through HVI approaches provides a safe and mild approach to chlorinated cyclic compounds.

#### Nitrogen nucleophiles

**Scheme 23:**
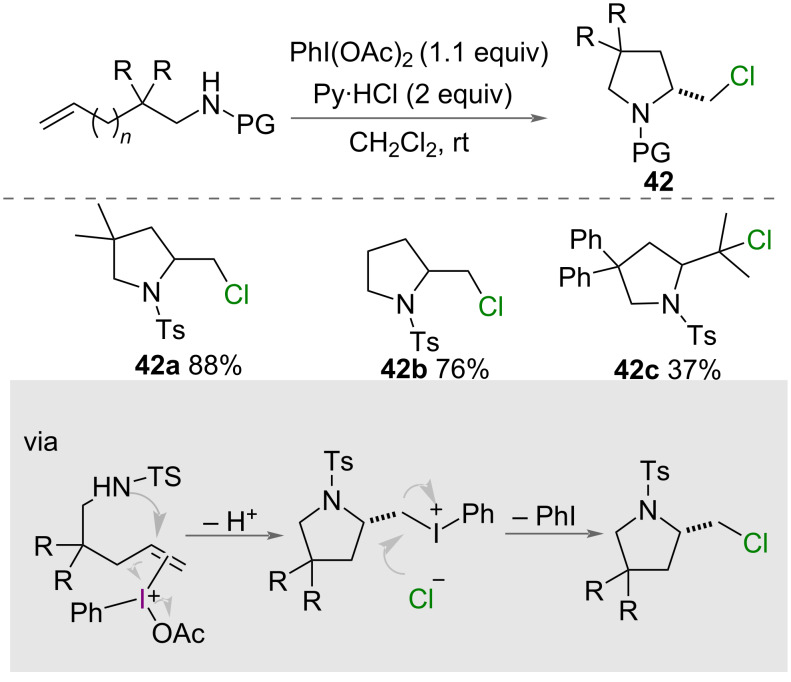
Intramolecular chlorocyclisation of unsaturated amines.

Intramolecular chlorocyclisation promoted by PhI(OAc)_2_ was reported by Liu and Li in 2014 alongside their intramolecular fluorocyclisation (Scheme 23) [30]. The authors reported the formation of 5- and 6-membered chlorinated azaheterocycles **42** from unsaturated amines, using PhI(OAc)_2_ as an oxidant and pyridinium chloride as a chlorine source. Substrates with a range of substituents on the alkyl chain were cyclised in good yields, yet introduction of substituents on the alkene led to a reduction of yield. The authors proposed a standard mechanism for the reaction (Scheme 23) in which PhI(OAc)_2_ activates the alkene, intramolecular attack of nitrogen forms the cyclised intermediate and a chloride ion displaces iodobenzene.

**Scheme 24:**
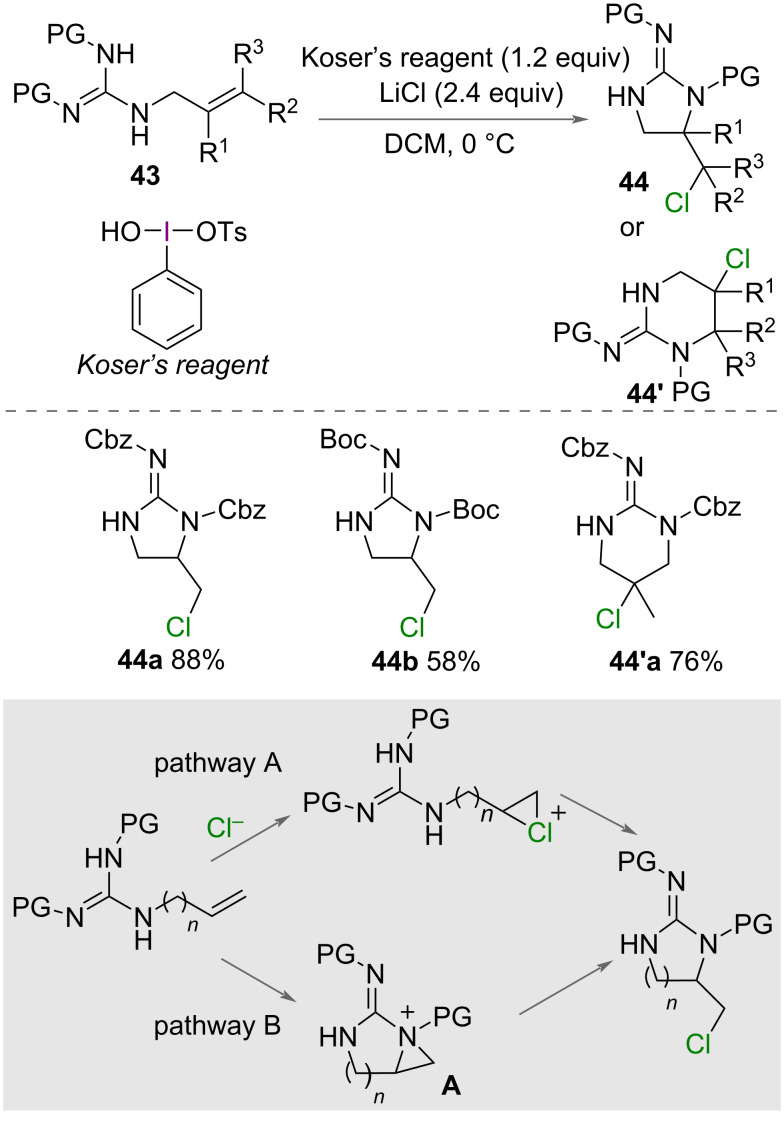
Synthesis of chlorinated cyclic guanidines **44**.

In 2015, Cariou, Dodd and co-workers reported the synthesis of chlorinated cyclic guanidines **44** (Scheme 24) [47]. Using Koser’s reagent as an oxidant with LiCl as a source of chloride, a range of unsaturated guanidines **43** were cyclised forming 5- or 6-membered rings in good yields. The change in ring size was proposed to be due to the position of the positive charge on the activated alkene. The carbon with the higher substitution has the greater positive charge and therefore undergoes nucleophilic attack by nitrogen. The authors proposed two possible mechanisms for the reaction (Scheme 24). Firstly, a chloronium ion is generated by HVI and LiCl followed by intramolecular nucleophilic attack by nitrogen to form the heterocycle. Secondly, oxidation of the unsaturated guanidine forms an intermediate aziridinium **A** with subsequent nucleophilic attack by chloride to form the product.

**Scheme 25:**
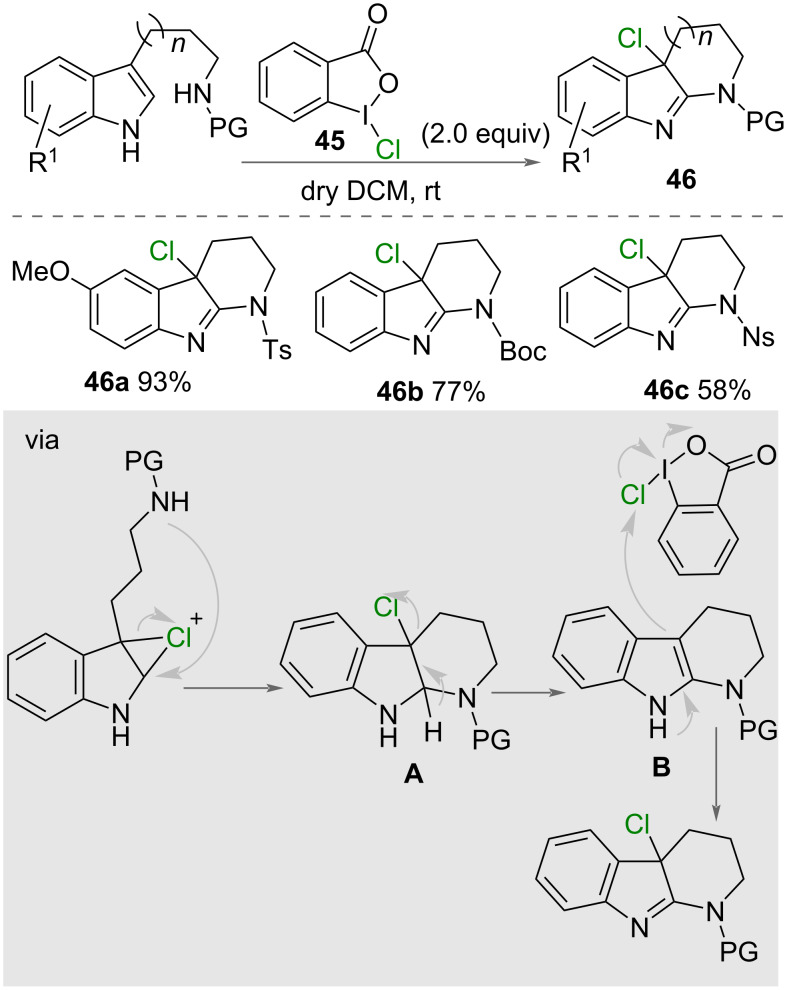
Synthesis of chlorinated pyrido[2,3-b]indoles **46**.

A metal-free chlorocyclisation of indole derivatives was reported by Yu and co-workers in 2019 (Scheme 25) [45]. The authors reported the use of 1-chloro-1,2-benziodoxol-3-one (**45**) as a single reagent to form 6- or 7-membered rings under mild conditions in DCM at room temperature. A range of substituents on the aromatic ring were tested with electron-withdrawing groups resulting in lower yields compared to electron-donating groups. The proposed mechanism for the reaction involved formation of a chloronium ion and nucleophilic attack from nitrogen on the less sterically-hindered carbon, forming a cyclic intermediate **A** (Scheme 25). Chloride is subsequently eliminated with formation of an enamine **B** that reacts with a second equivalent of 1-chloro-1,2-benziodoxol-3-one (**45**) to afford the product **46**.

#### Oxygen nucleophiles

**Scheme 26:**
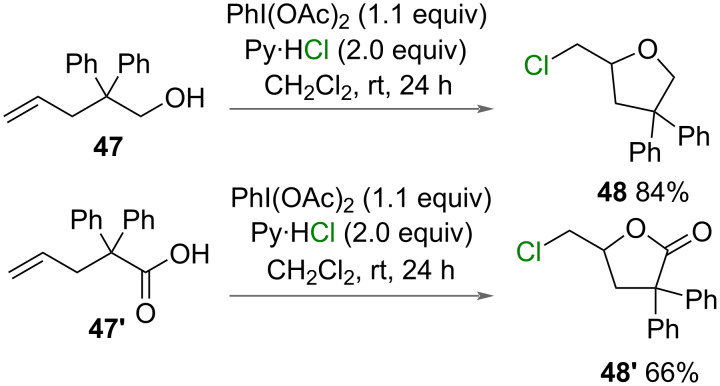
Chlorolactonization and chloroetherification reactions.

Liu and Li reported the chlorolactonization and chloroetherification (Scheme 26) of 2,2-diphenylpent-4-enoic acid (**47’**) and 2,2-diphenyl-4-penten-1-ol (**47**), respectively, using PhI(OAc)_2_ as an oxidant and pyridinium chloride as a chlorine source. This was an expansion of their scope that used nitrogen nucleophiles [30].

**Scheme 27:**
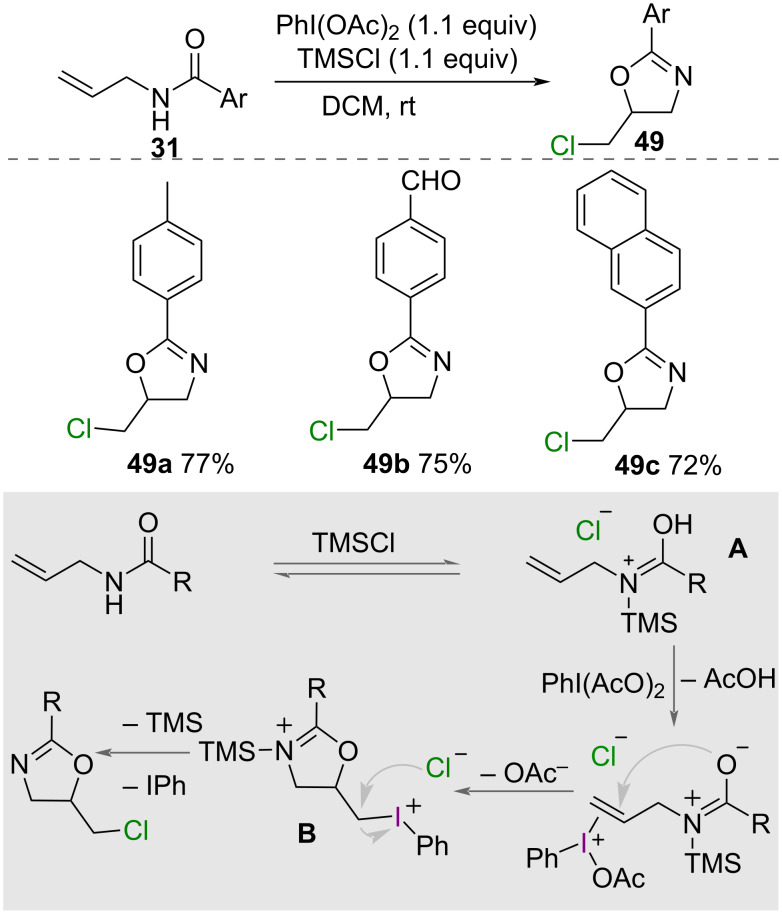
Proposed mechanism for the synthesis of chloromethyl oxazolines **49**.

In 2015, Li and co-workers reported the synthesis of chloromethyloxazolines **49** [48] (Scheme 27). Using PhI(OAc)_2_ as an oxidant and TMSCl as a source of chloride and activator, a range of *N*-allyl carboxamides **31** were successfully cyclised, forming 5-chloromethyl-2-aryloxazolines **49** in good yields. A mechanism for the reaction was proposed by the authors (Scheme 27), whereby a TMS-adduct **A** of the amide is formed, alkene activation and cyclisation from oxygen forms the cyclised intermediate **B**, then displacement of PhI by chloride gives the product.

**Scheme 28:**
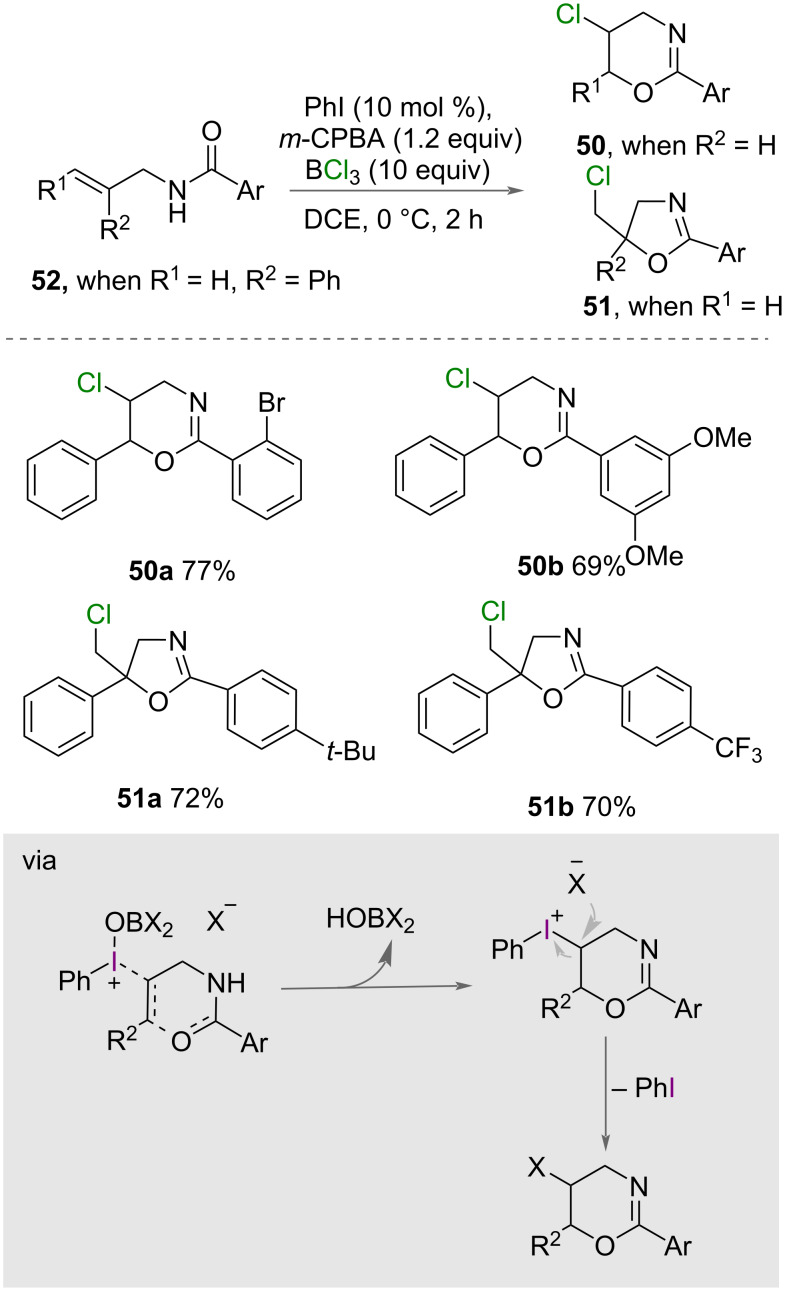
Oxychlorination to form oxazine and oxazoline heterocycles promoted by BCl_3_.

Chai, Jiang, Zhu and co-workers reported the synthesis of various halogenated 1,3-oxazine **50** and 2-oxazoline derivatives **51** using boron trihalides as the halogen source [6,49]. They found that the choice of halogen source influences the reaction outcomes. With the use of BCl_3_ (Scheme 28), *N*-cinnamylbenzamides **52** were transformed to give the corresponding chlorinated dihydro-[1,3]-oxazines **50** in good to excellent isolated yields [49]. When various substituted *N*-(2-phenylallyl)benzamides (**52**) were tolerated, it led to the formation of chlorinated 2-oxazolines **51** in good to excellent yields. When BF_3_ was used as the halogen source in the author’s previous work (Scheme 21) [6], it led to the formation of different products compared to when BCl_3_ was utilized, suggesting a different mechanism is operative.

### Hypervalent iodine-mediated bromocyclisation

Bromocyclisation promoted by HVI reagents allows for a mild, metal-free synthesis of various cyclic functional groups and avoids the use of highly toxic and corrosive bromine. Approaches using this approach are outlined below.

#### Nitrogen nucleophiles

**Scheme 29:**
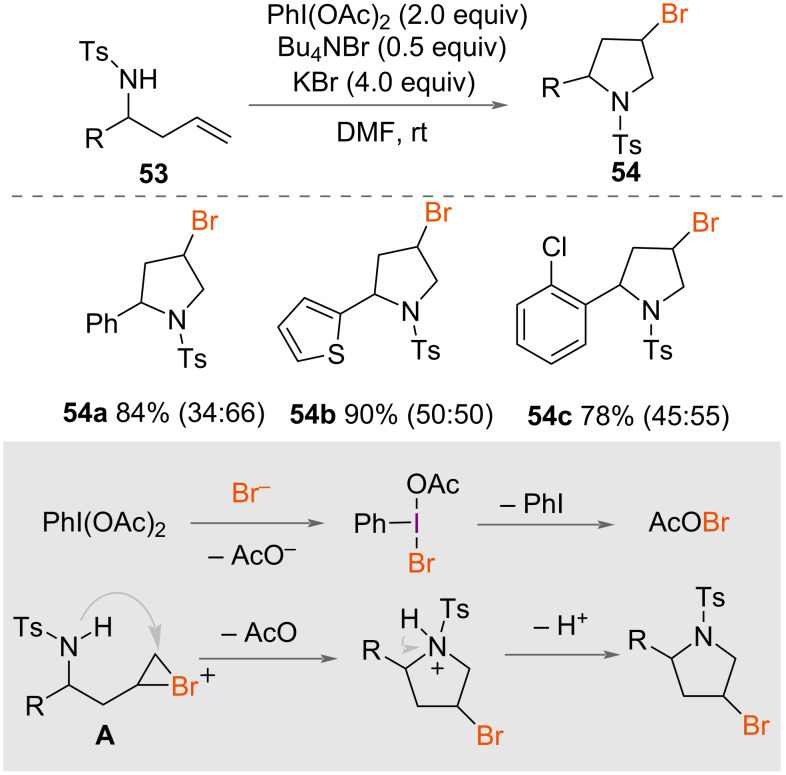
Aminobromocyclisation of homoallylic sulfonamides **53**. The *cis*:*trans* ratios based on the ^1^H NMR of the corresponding products are given in parentheses.

In 2007, aminobromocyclisation of homoallylic sulfonamides **53** was reported by Fan, Wang and co-workers (Scheme 29) [50]. Using PhI(OAc)_2_ as an oxidant with KBr as the bromine source and Bu_4_NBr as a reaction promoter, racemic brominated pyrrolidines **54** were synthesised from a range of homoallylic sulfonamides **53** in excellent yields under mild conditions at room temperature. A mechanism was suggested by the authors (Scheme 29), whereby ligand exchange on PhI(OAc)_2_ with a bromide ion forms unstable PhIOAcBr. Elimination of bromoacetate then gives a reactive electrophilic bromine source, which forms a bromonium intermediate **A** after reaction with the alkene. Intramolecular nucleophilic attack from nitrogen forms the product **54**.

**Scheme 30:**
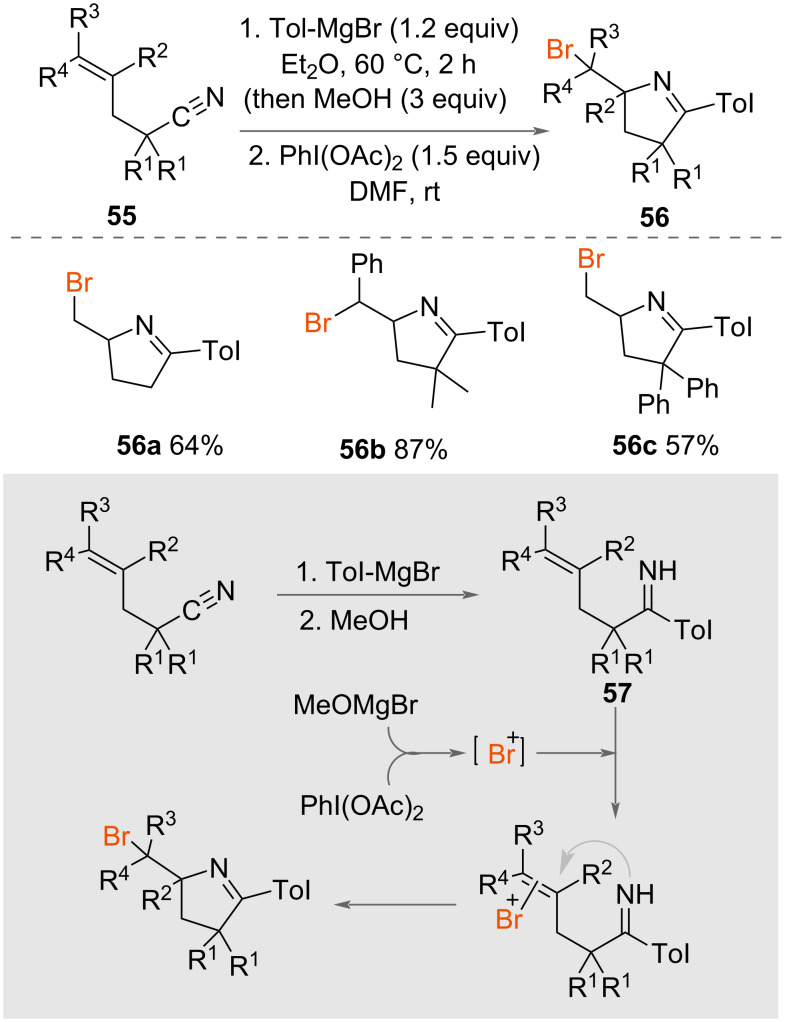
Synthesis of cyclic imines **45**.

Chiba and co-worker reported the synthesis of cyclic imines using a one-pot protocol involving Grignard addition to a cyano group followed by PhI(OAc)_2_ (Scheme 30) [51]. The authors used *p*-tolylmagnesium bromide for both the arylation of the unsaturated carbonitriles **55** and as a bromide source. Bromocyclisation was achieved using PhI(OAc)_2_, which formed a range of 5- and 6-membered bromomethyl cyclic imines **56** in good yields from unsaturated imines **57**.

**Scheme 31:**
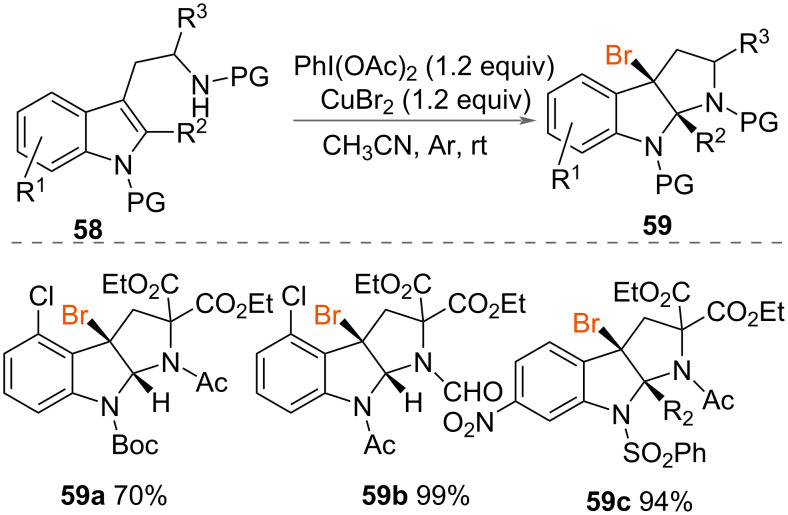
Synthesis of brominated pyrrolo[2,3-*b*]indoles **59**.

Xia and co-workers reported the bromocyclisation of indole derivatives **58** (Scheme 31) using PIDA and CuBr_2_ as the oxidant and bromide source, respectively [52]. Racemic pyrrolo[2,3-*b*]indoles **59** were synthesised in up to quantitative yields under mild reaction conditions at room temperature. A range of other indole derivatives were cyclised in similarly good yields demonstrating the scope of the reaction.

**Scheme 32:**
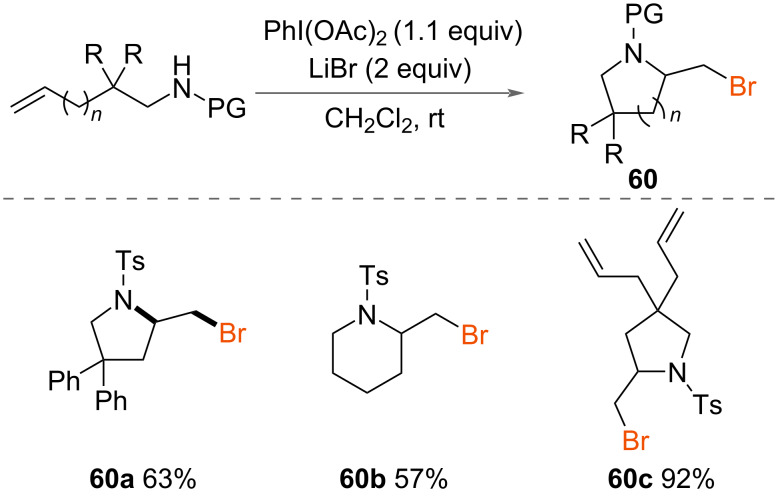
Bromoamidation of alkenes.

Li and Liu reported the bromoamidation of alkenes in 2014 (Scheme 32) [30]. Using PhI(OAc)_2_ as an oxidant and LiBr as a source of bromine, a range of unsaturated amines were successfully cyclised to from 5- and 6-membered aza-heterocycles **60** under mild conditions at room temperature.

**Scheme 33:**
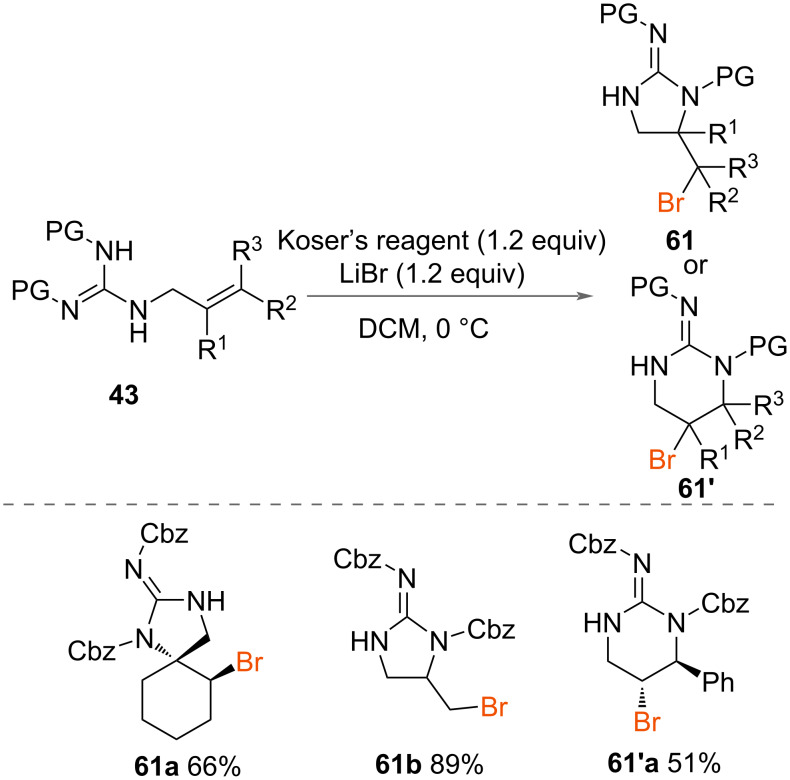
Synthesis of brominated cyclic guanidines **61** and **61’**.

Cariou, Dodd and co-workers reported in 2015 the synthesis of brominated cyclic guanidines **61** (Scheme 33) alongside their chlorinated cyclic guanidines **44** (vide supra) [47]. Koser’s reagent was employed with LiBr to form 5- and 6-membered brominated cyclic guanidines **61** and **61’** in good yields from allylic guanidines **43**. A range of substrates with substituents on the terminal alkene were investigated, which were successfully cyclised to brominated 5- or 6-membered rings in good yields.

**Scheme 34:**
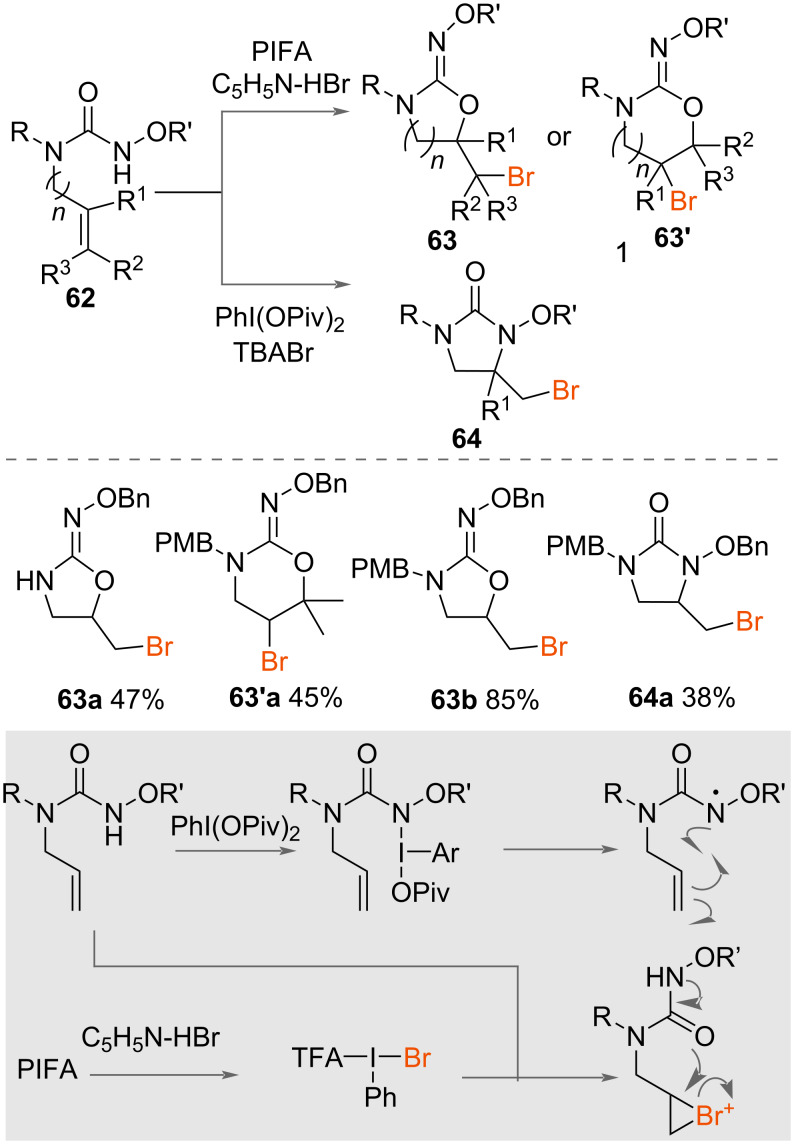
Intramolecular bromocyclisation of *N*-oxyureas.

The intramolecular bromocyclisation of *N*-oxyureas was also reported by Cariou and co-workers in 2019 (Scheme 34) [53]. From the same starting material, the authors reported the synthesis of both oxazolidinone oximes **63** and *N*-hydroxylated ureas **64** depending on the reagent system used. Formation of oxazolidinone oximes **63** occurred using PhI(OCOCF_3_)_2_ (PIFA) as an oxidant with pyridine·HBr and the MgO additive. The oxybromocyclisation of a range of unsaturated *N*-alkoxyureas **62** occurred rapidly in 10 minutes at room temperature in acetonitrile with good yields. Formation of the *N*-hydroxylated ureas **64** occurred using PhI(OPiv)_2_ and TBABr, with MgO as an additive to trap acetic acid. Aminobromocylization of a range of unsaturated *N*-alkoxyureas **62** was less successful with longer reactions times up to 1 hours required and poorer yields afforded. The rationale for the difference in mechanism was attributed to the oxycyclisation to yield oxazolidinone oximes **63** occurring through an ionic mechanism, whereas the aminocyclisation takes place through a radical manifold, a difference that is triggered by the difference in HVI reagent used.

**Scheme 35:**
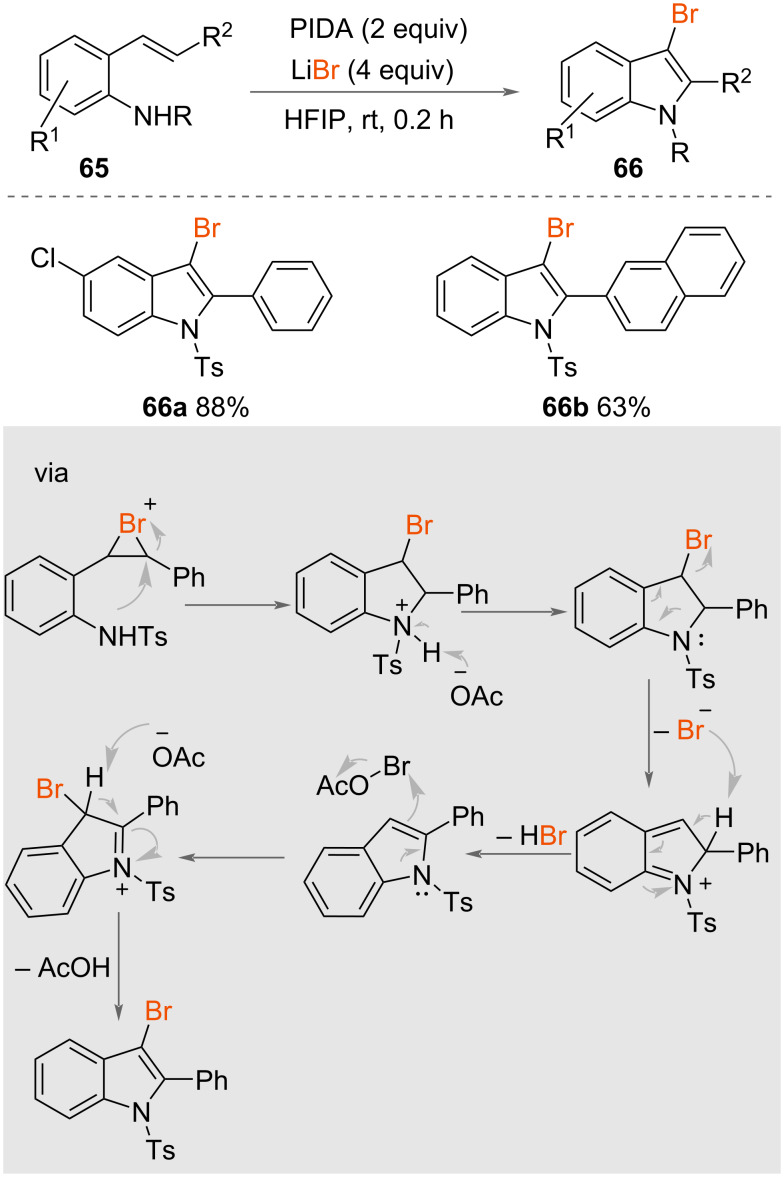
The formation of 3-bromoindoles.

In 2023, Du and co-workers reported a method for synthesizing 3-bromoindoles via a cascade oxidative cyclisation–halogenation encompassing oxidative C−N/C−Br bond formation, and utilising phenyliodine(III) diacetate (PIDA) in combination with LiBr in HFIP (Scheme 35) [54]. The reaction of 2-alkenylanilines **65** with PIDA and LiBr resulted in the successful synthesis of various 3-bromoindoles **66** in high yields obtained under the optimised reaction conditions, highlighting the efficiency of the synthetic protocol. The proposed mechanism suggests that the reactive AcO–Br species is formed in situ from the reaction of PIDA and LiBr.

#### Oxygen nucleophiles

**Scheme 36:**
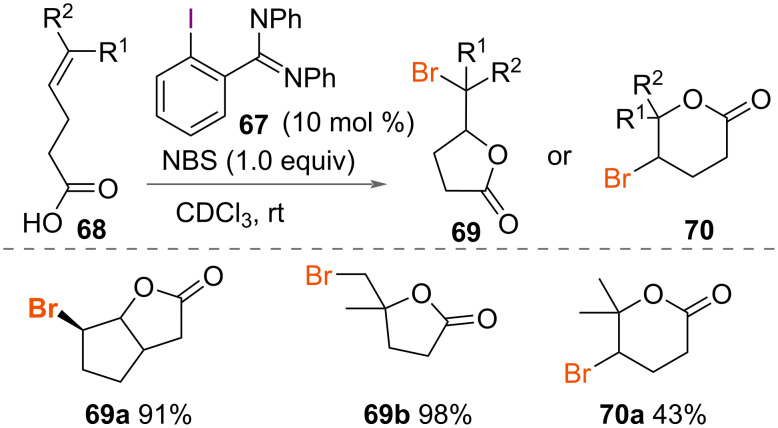
Bromolactonisation of unsaturated acids **68**.

A novel use of HVI reagents that promotes bromocyclization was reported by Braddock and co-workers in 2006 (Scheme 36) [55]. The authors reported the use of a bromoiodinane, formed in situ from *ortho*-substituted amidine iodobenzene **67** and *N*-bromosuccinimide (NBS), which promoted the intramolecular bromolactonisation of unsaturated acids **68**. The authors investigated the oxidation using a variety of *ortho*-substituted iodobenzenes. Increasing the nucleophilicity of the groups at the *ortho*-substituted positions of iodobenzene gave increased yields of cyclised product. Both 5- and 6-membered bromolactone products **69** and **70** were formed with 5-*exo* ring closure preferred.

**Scheme 37:**
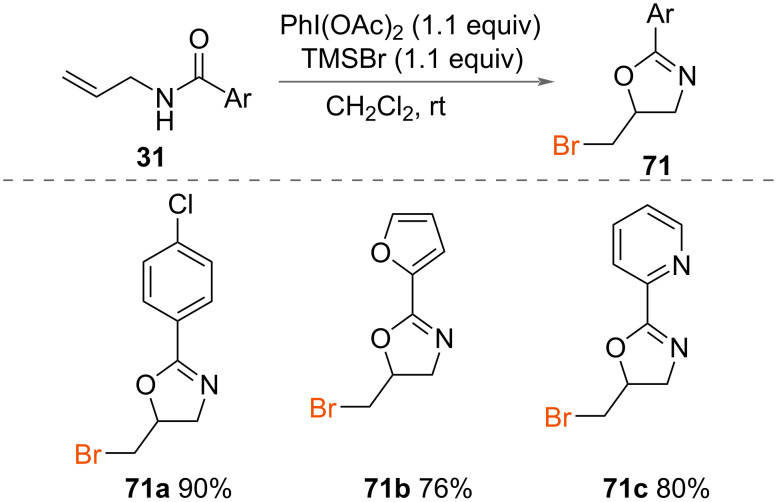
Synthesis of 5-bromomethyl-2-oxazolines.

In addition to the synthesis of 5-chloromethyl-2-oxazolines **49**, Li and co-workers reported the preparation of 5-bromomethyl-2-oxazolines **71** (Scheme 37) [48]. Treatment of a range of *N*-allyl carboxamides **31** with PhI(OAc)_2_ and TMSBr formed 5-bromomethyl-2-oxazolines **71** in excellent yields.

**Scheme 38:**
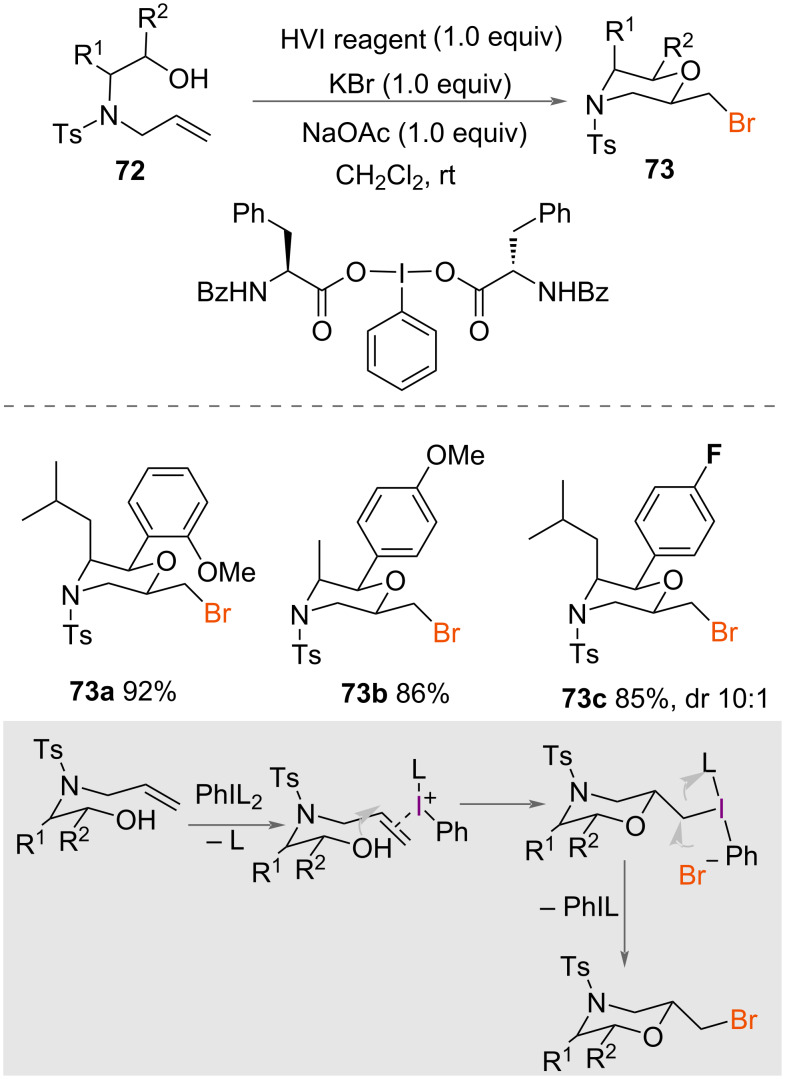
Synthesis of brominated chiral morpholines.

In 2015, Wang and co-workers reported the bromocyclisation of allylamino alcohols **72** to give chiral morpholines **73** (Scheme 38) [56]. Using an amino acid-derived chiral HVI reagent with KBr and NaOAc, a range of chiral 2,3,6-trisubstituted bromomethylmorpholines **73** were synthesised in excellent yields and diastereoselectivities that ranged from nothing to excellent depending on the substrate. The enantioselectivity of the reaction was not measured. The authors suggested a mechanism for the reaction (Scheme 38) in which activation of the alkene and intramolecular attack of oxygen through a 6-*exo*-*trig* mechanism followed by S_N_2 reaction with bromide eliminates the chiral aryliodide to form the product **73**.

**Scheme 39:**
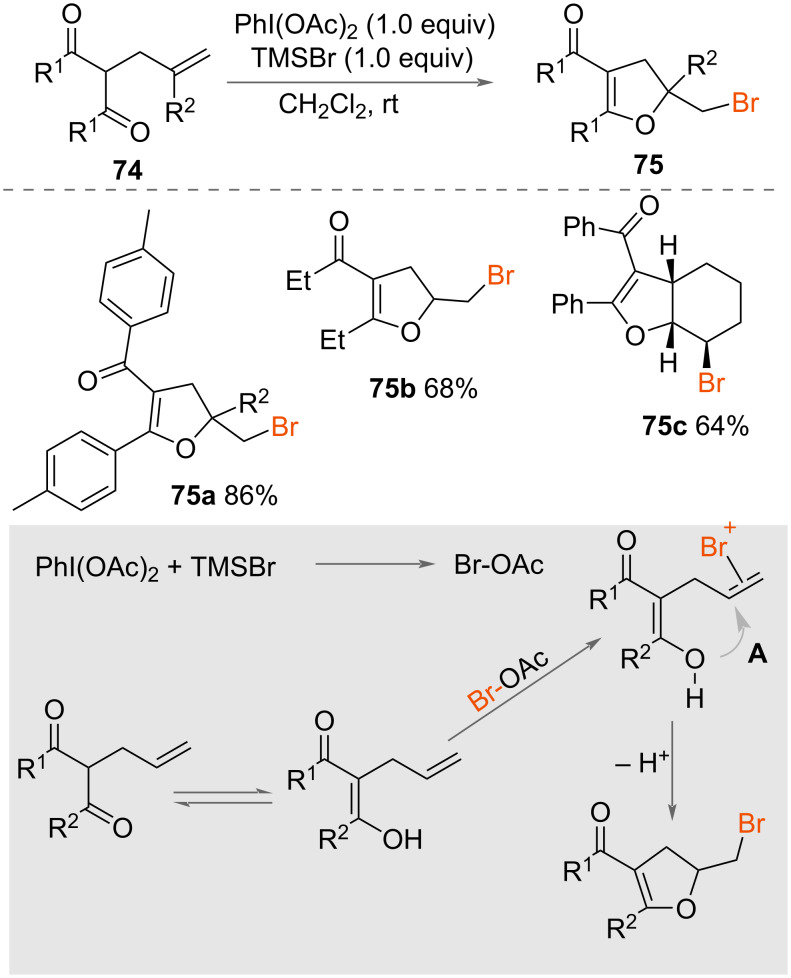
Bromoenolcyclisation of unsaturated dicarbonyl groups.

In 2018, Liu, Ling and co-workers reported bromoenol-cyclisation of unsaturated dicarbonyls **74** (Scheme 39) [57]. Using PhI(OAc)_2_ as an oxidant and TMSBr as a source of bromine and reaction promoter, a range of bromomethyldihydrofurans **75** were cyclised in good yields. Both 5- and 6-membered rings were formed, with homologation of the unsaturated chain. The authors initially proposed two alternative mechanisms for the reaction (Scheme 39). Either the reaction of PhI(OAc)_2_ (PIDA) with TMSBr generates BrOAc, which forms a bromonium ion **A** with the alkene, followed by intramolecular nucleophilic attack of oxygen to form the cyclic product **75**. Alternatively, the alkene could be activated after coordination to PhI(OAc)_2_, then intramolecular nucleophilic attack from oxygen and nucleophilic attack by bromide forms the final product. NMR studies of PIDA and the substrate indicated that there was no interaction between them, thereby discounting this second pathway and thereby providing support for the former pathway (Scheme 39).

**Scheme 40:**
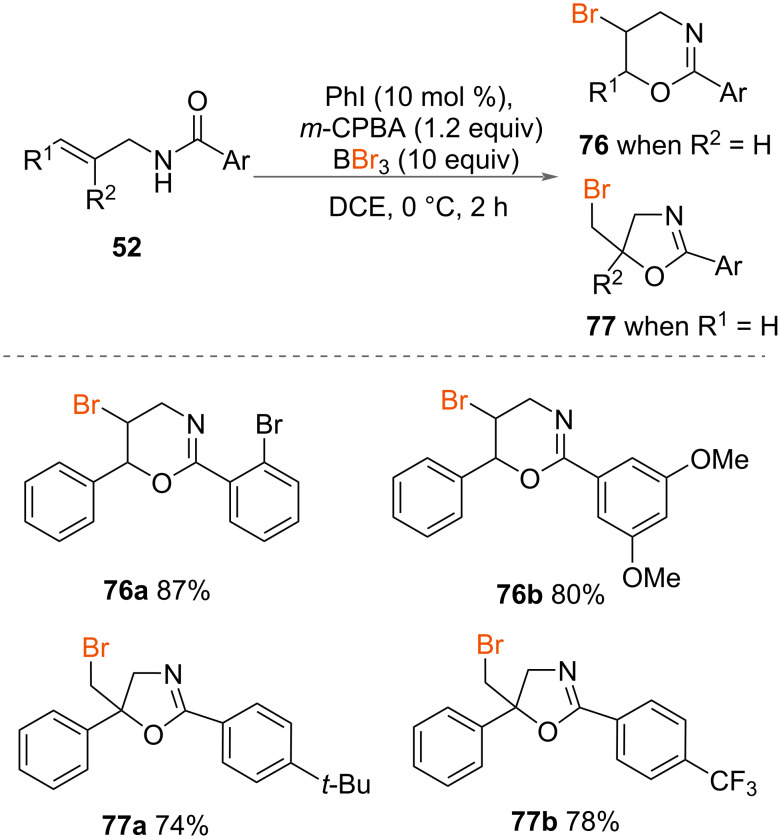
Brominated oxazines and oxazolines with BBr_3_.

In 2023, Chai, Jiang, Zhu and co-workers included the oxybromination of alkenes to form brominated dihydro-[1,3]-oxazines **76** and 2-oxazoline **77** derivative (Scheme 40) [6,49], alongside their chlorination examples. Optimized reaction conditions were developed with BBr_3_ as bromide source and activating reagent, which led to the formation of the brominated oxazines **76** and 2-oxazoline **77** in very good to excellent yields. The authors found that when substituted *N*-(2-phenylallyl)benzamides **52** were tolerated, it led to the formation of brominated 2-oxazolines in excellent yields. The structures were also assigned by X-ray crystallography.

#### Sulfur nucleophiles

**Scheme 41:**
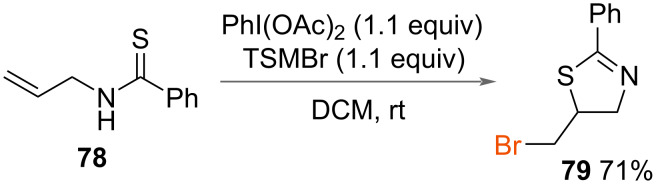
Synthesis of 5-bromomethtyl-2-phenylthiazoline.

In 2015, Li and co-workers reported the synthesis of 5-bromomethyl-2-phenylthiazoline (**79**, Scheme 41) [48]. Sulfur was used as the internal nucleophile instead of nitrogen, as previously reported by the authors in the formation of oxazolines. Using PhI(OAc)_2_ with TMSBr as an oxidant and source of bromine respectively, *N*-allylbenzothioamide (**78**) was cyclised to form 5-bromomethtyl-2-phenylthiazoline (**79**) in a good yield.

### Hypervalent iodine-mediated iodocyclisation

Reactions involving iodocyclisation mediated by HVI compounds are less prevalent compared to other halocyclisations. The use of PhI(OAc)_2_ with KI or TMSI as the iodide source has been reported to promote iodocyclisation in unsaturated compounds with internal nucleophiles.

#### Nitrogen nucleophiles

**Scheme 42:**
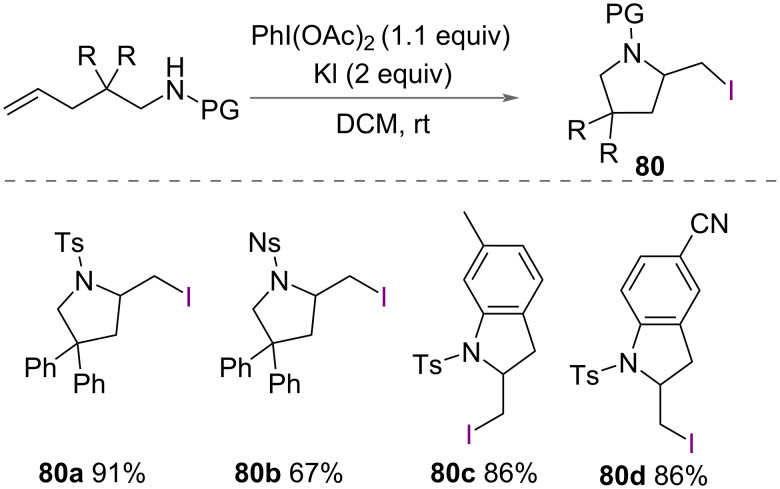
Intramolecular iodoamination of unsaturated amines.

In addition to both chloro- and bromoaminations, Liu and Li reported intramolecular iodoamidiation of unsaturated amines in 2014 (Scheme 42) [30]. Using PIDA as an oxidant and KI as a source of iodide, iodinated pyrrolidines **80** were synthesized in excellent yields under mild conditions of CH_2_Cl_2_ at room temperature. A range of unsaturated amines were successfully cyclised to form 5-membered rings. Lower yields were observed with substituted alkenes. A mechanism was proposed by the authors identical to both chloro- and bromoaminations previously reported.

**Scheme 43:**
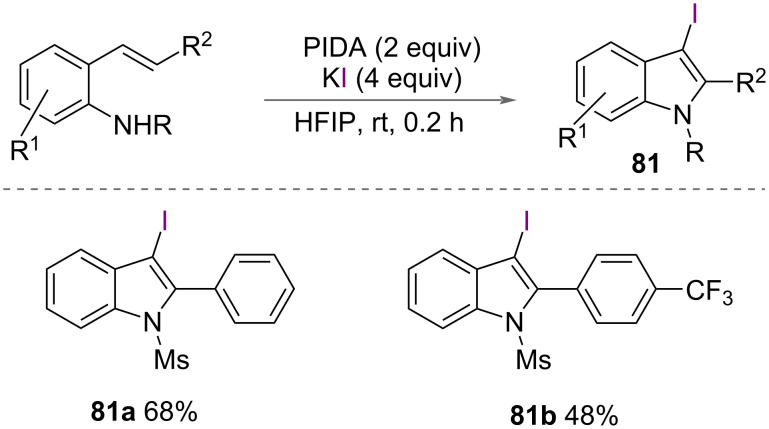
Formation of 3-iodoindoles.

Du and co-workers described the formation of 3-iodoindoles **81** in their 2023 report that also demonstrated the formation of 3-bromoindoles **66** (Scheme 43) [54]. In this instance, KI was used as the iodide source with PIDA in HFIP. The mechanism proposed was the same as that for the bromoindoles (Scheme 35).

#### Oxygen nucleophiles

**Scheme 44:**
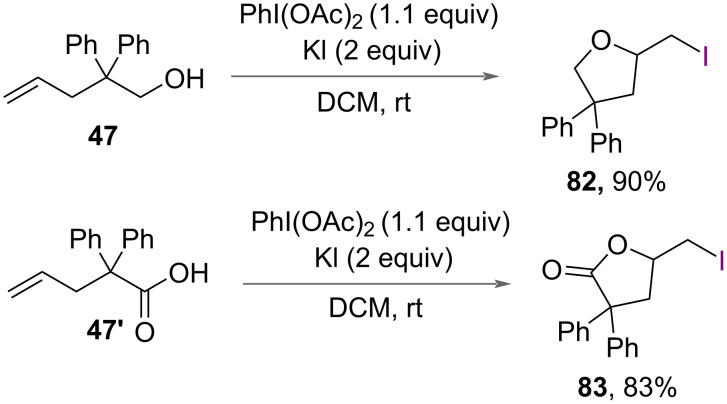
Iodoetherification of 2,2-diphenyl-4-penten-1-carboxylic acid (**47’**) and 2,2-diphenyl-4-penten-1-ol (**47**).

In addition to chloroamidation and chlorolactonization, iodoetherification of 2,2-diphenyl-4-penten-1-carboxylic acid (**47'**) and 2,2-diphenyl-4-penten-1-ol (**47**) was reported by Liu and Li in 2014 (Scheme 26) [30]. The authors reported using PhI(OAc)_2_ and KI in the synthesis of iodonated γ-butyrolactone **83** and iodomethyltetrahydrofuran **82** in excellent yields (Scheme 44).

**Scheme 45:**
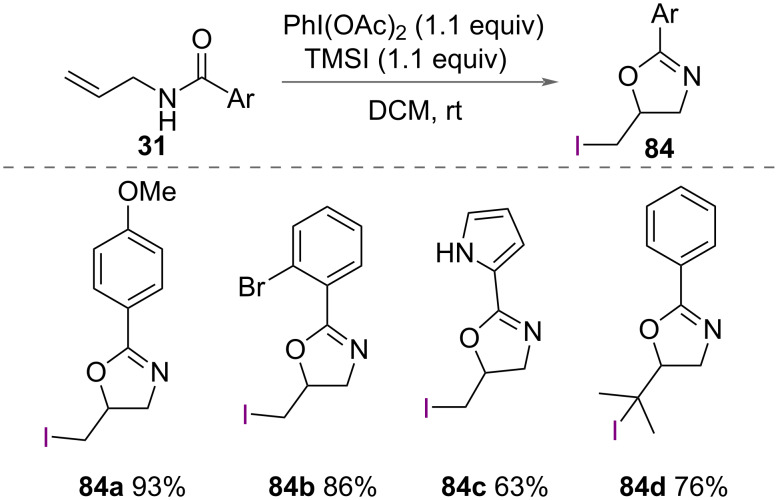
Synthesis of 5-iodomethyl-2-oxazolines.

Li and co-workers reported the synthesis of 5-iodomethyl-2-aryloxazolines **84** in addition to the synthesis of 5-chloromethyl-2-aryloxazolines **49** and 5-bromomethyl-2-aryloxzaolines **71** (Scheme 27) [48]. Treatment of a *N*-allyl carboxamides **31** with PhI(OCOCH_3_)_2_ as an oxidant and TMSI as a source of iodide formed a range of 5-iodomethyl-2-aryloxazolines **84** in good yields (Scheme 45).

**Scheme 46:**
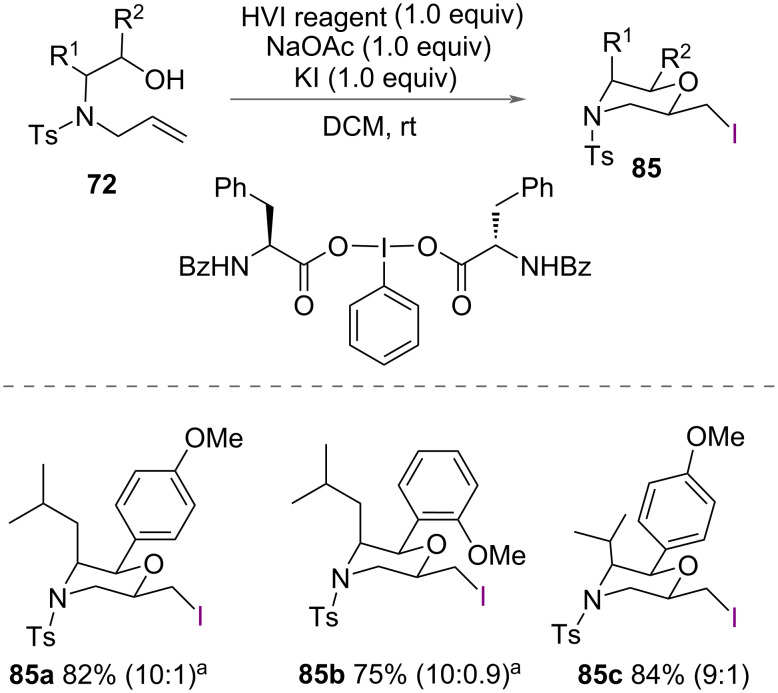
Synthesis of chiral iodinated morpholines. ^a^From the ʟ-form of the amino acid starting material. The dr values were determined by ^1^H NMR given in parentheses.

In addition to the synthesis of brominated morpholines, Wang and co-workers reported the synthesis of chiral iodinated morpholines **85** (Scheme 46) [56]. Using an amino acid-derived chiral iodine(III) reagent with KI and NaOAc, a range of allylamino alcohols **72** were cyclised in excellent yields, again without reporting the enantioselectivity of the reaction.

**Scheme 47:**
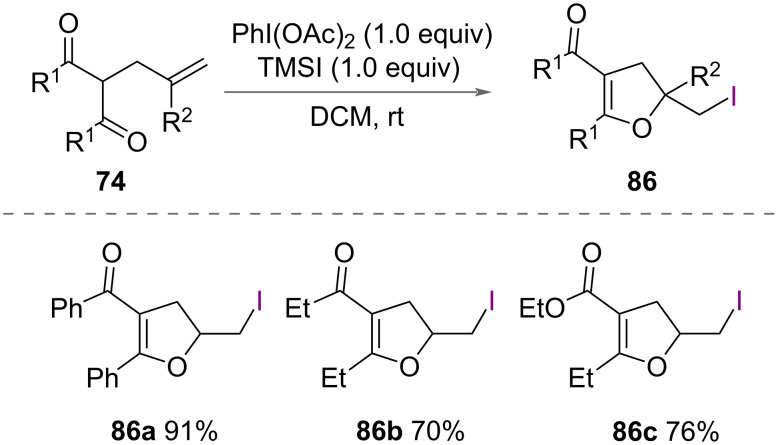
Iodoenolcyclisation of unsaturated dicarbonyl compounds **74**.

As well as bromoenol cyclisation (Scheme 39), Liu, Ling and co-workers reported iodoenolcyclisation of unsaturated dicarbonyl compounds **74** in 2018 (Scheme 47) [57]. Using PhI(OAc)_2_ and TMSI, a range of polysubstituted iodomethyldihydrofurans **86** were successfully synthesised in good yields. From NMR measurements, the authors proposed the formation of an iodonium intermediate.

#### Sulphur nucleophiles

**Scheme 48:**
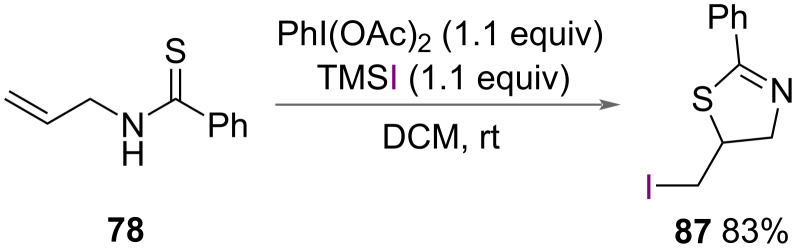
Synthesis of 5-iodomethtyl-2-phenylthiazoline (**87**).

Li and co-workers reported the synthesis of 5-iodomethyl-2-phenylthiazoline (**87**) in 2015 in addition to the synthesis of 5-bromomethtyl-2-thiazoline (Scheme 41), using sulfur as an internal nucleophile (Scheme 48) [48]. Using PhI(OAc)_2_ with TMSI, *N*-allylbenzothioamide (**78**) was cyclised to form **87** in excellent yield.

## Conclusion

The HVI-mediated halocyclization of alkenes is an important approach that yields a broad variety of heterocycles under mild and efficient oxidative conditions. The internal nucleophile, length of the carbon chain and halide can be designed such that a very broad range of 5-, 6- and 7-membered heterocycles can be accessed. The identity of the HVI reagent has also shown to dictate the regioselectivity of the reaction. Future research efforts should focus on further developing access to new heterocycles, as well as designing better systems to incorporate high levels of diastereoselectivity and enantioselectivity into chiral halogenated heterocycles.

## Data Availability

Data sharing is not applicable as no new data was generated or analyzed in this study.
